# Women and insurance pricing policies: a gender-based analysis with GAMLSS on two actuarial datasets

**DOI:** 10.1038/s41598-024-52959-8

**Published:** 2024-02-08

**Authors:** Giuseppe Pernagallo, Antonio Punzo, Benedetto Torrisi

**Affiliations:** 1 Department of Economics and Statistics “Cognetti de Martiis”, University of Turin, Lungo Dora Siena, 100A, 10153 Turin, Italy; 2 Department of Economics and Business, University of Catania, Corso Italia 55, 95129 Catania, Italy

**Keywords:** Engineering, Mathematics and computing

## Abstract

In most of the United States, insurance companies may use gender to determine car insurance rates. In addition, several studies have shown that women over the age of 25 generally pay more than men for car insurance. Then, we investigate whether the distributions of claims for women and men differ in location, scale and shape by means of the GAMLSS regression framework, using microdata provided by U.S. and Australian insurance companies, to use this evidence to support policy makers’ decisions. We also develop a parametric-bootstrap test to investigate the tail behavior of the distributions. When covariates are not considered, the distribution of claims does not appear to differ by gender. When covariates are included, the regressions provide mixed evidence for the location parameter. However, for female claimants, the spread of the distribution is lower. Our research suggests that, at least for the contexts analyzed, there is no clear statistical reason for charging higher rates to women. While providing evidence to support unisex insurance pricing policies, given the limitations represented by the use of country-specific data, this paper aims to promote further research on this topic with different datasets to corroborate our findings and draw more general conclusions.

## Introduction

The research question of this paper stems from a popular belief, common in many countries. There are numerous quips regarding female drivers, who are often depicted as less skilled drivers than men. In Italy, for example, men usually say “donne al volante, pericolo costante”, which can be (approximately) translated as “women driving, peril thriving”. Albeit the issue may seem frivolous, it assumes great importance from the perspective of insurers, risk analysts and policy makers. If women were indeed worse customers for insurers, gender would represent an important variable to model insurance-related data. This study aims to provide evidence to determine whether insurers are statistically justified in treating women and men differently using claims data.

We have two main research objectives. Firstly, we look for the best model for the loss distribution (a largely debated issue in literature) and we investigate whether gender makes differences in some aspects of the distribution such as, for example, location or scale. Secondly, we evaluate whether gender affects the magnitude of losses, controlling for other available covariates. In particular, we give emphasis to the largest claims (the right tail of the loss distribution), which are of relevant importance for insurance companies.

In summary, our contribution is mainly empirical in nature, but also partly methodological. Empirically, we aim to provide evidence to answer the important policy question of whether gender is a relevant variable for insurers. These results are limited by the use of available data, but have important economic value for both insurers and policy makers (see “Potential limitations” for a discussion). On the other hand, we also contribute to the methodological literature by proposing the use of many statistical models neglected in similar works and introducing a bootstrap test to test for differences between groups in the tails of their distributions.

There are several studies related to the issue. Sivak and Schoettle^1^ study the representativeness of gender in six different crash scenarios. Even though the results may be influenced by different factors, the authors find that, in some scenarios, male-to-male crashes tend to be underrepresented, whereas female-to-female crashes tend to be over-represented.

A study of prominent interest for insurers was carried out by Massie et al.^2^ on passenger-vehicle travel data. The authors find that elevated crash involvement rates per vehicle-mile of travel are registered for young individuals (aged 16–19) and old drivers (75 and over). Men are more likely to experience a fatal crash whereas women are more frequently involved in injury crashes and in all police-reported crashes. Santamariña-Rubio et al.^3^ provide contrasting evidences: first, the authors find the presence of an interaction effect between gender and age in road traffic injury risk; second, in some age groups men show excess risk compared to women, while in others they observe the opposite, with a dependence on the severity of the injury and the mode of transport.

Several studies have shown that, in general, women drive more cautiously than men^2,4–7^. Moreover, as documented in Regev et al.^8^, p. 131, “*driver’s age and gender have also been shown to affect the severity of crash outcomes (i.e. the risk of fatal injury given a crash)*”, with a higher likelihood to be exposed to fatal injuries in a crash for male and elderly drivers than female and young drivers^9,10^.

The theme of this paper is merely economic: if gender affects the likelihood of being involved in a crash or the severity of a car accident (and therefore economic losses for a company), then insurance companies may require different rates. The debate is still open. For example, a recent article of the HuffPost (Car Insurance Companies Charge Women Higher Rates Than Men Because They Can, by Elaine S. Povich, 2019, HuffPost) revealed that several studies in 2017 and 2018 showed that women over 25 generally pay more than men for auto insurance. As reported in the article, in many cases (and for the same policy) women paid $500 more than men for no reason other than their gender. The European Union, as reported by The Guardian (How an EU gender equality ruling widened inequality, by Patrick Collinson, 2019, The Guardian), introduced rules to avoid gender discrimination by car insurance companies, a practice detrimental for the principle of unisex pricing. One may argue that the variable “gender” is fully controlled by legislators, but this is not true for many relevant geographical contexts. As reported by the Business Insider (Car insurance rates are going up for women across the US—here’s where they pay more than men, by Shayanne Gal and Tanza Loudenback, 2019, Business Insider), in 44 US states insurance companies can use gender to determine a driver’s car insurance rate, whereas only the states of California, Hawaii, Massachusetts, Montana, North Carolina, and Pennsylvania have banned the practice. Therefore, the present study is of prominent interest for legislators of many states.

Risk classification is necessary in the insurance industry. Hence, some sort of differentiation is needed to operate optimally in the market, but such decisions require a “fair justification”^11^. As analysts, this means that gender-based price discrimination should be statistically motivated. Loss or claims data have been treated in literature generally without differentiating by gender (which is surprising given the huge quantity of studies in the field). These studies (see “Literature review”) consider many aspects of the data, from the distributional properties to predictive models. With this paper we want to check whether similar results hold when we separate data based on the claimant’s gender, using two important datasets provided in R packages.

We believe that our study is of interest for five main reasons. First, to our knowledge this is the first study that implements distribution fitting to claims data separating by gender. Studies in this field are generally concerned only with finding the best model for the whole distribution. Second, our empirical analysis shows the good performance of many statistical models neglected in the field. Third, we introduce a new parametric test to check whether VaR computed for females data differs from VaR computed for males data. The test has been conceived for our case study but can be used also in different contexts. Fourth, we show the power of GAMLSS modelling when dealing with asymmetric and/or non-mesokurtic data, or when a researcher aims to modify existing distributions, for example, via truncation or adjusting for zeros. Indeed, this approach can yield enormous benefits in modelling economic or financial data. Last but not least, we provide guidance for policy makers, encouraging the application of a fair pricing.

The paper is structured as follows. “Literature review” presents a review of the existing literature. “Data” describes the data used in the empirical analysis. “Methodology” illustrates the adopted statistical methodology. “Distribution fitting results” describes the results of the regression analysis when the available covariates are not included (hereafter often referred to as “distribution modelling/fitting”) where we also test for differences in the two distributions. “Regression results” shows the regression results when the available covariates are included in the analysis: we check whether gender is related to insurers’ claims, considering the whole distribution and the tail of the data. In “Potential limitations”, we discuss a series of shortcomings that could undermine the validity of our results. “Conclusions and policy implications” concludes the paper. Appendices (A, B, and C) are distributed as online supplementary material.

## Literature review

### Distribution modelling

Regarding the first research question of this paper, we need to understand whether the claims of females and males behave differently in distribution. It has been shown in many works that the distribution of insurance losses is generally heavy-tailed^12,13^, unimodal hump-shaped or multimodal^14–16^ and skewed^13,17,18^. Moreover, it is important to account for the positive support of the distribution^16,19–22^.

Among the many parametric models proposed in literature for the loss distribution^19^, Eling^18^ assesses the performance of the following classical distributions: Normal, Student’s *t*, hyperbolic, generalized hyperbolic, normal inverse Gaussian, variance gamma, gamma, Weibull, Cauchy, skew-normal, skew-*t*, logistic, log-normal, exponential, Pareto, chi-square and geometric. As pointed out by Eling^18^, the Pareto distribution is a relevant statistical model in catastrophe insurance to describe, especially, large losses, and many authors have used it as a starting framework for modelling losses and lifetime data, or in any context characterised by heavy-tailed distributions^23–25^. The more flexible family of the generalized Pareto distributions, albeit promising to fit insurance data, has not found the same favour by researchers, probably because estimation methods like the maximum likelihood and method-of-moments are undefined in some regions of the parameter space, making the fitting procedure a difficult routine^26^.

Recently, some authors have focused their attention on more sophisticated, but also more flexible, composite^14,24^, compound^16,22^ and mixture^20,27,28^ models. All these approaches share the common principle to combine the characteristics of two or more distributions, so modelling many aspects that a single distribution cannot represent.

We provide novelty to this already large stream of papers in different ways. Firstly, we fit renowned, but also less used, parametric models to important car insurance datasets. Secondly, we avoid the boundary bias issue^29,30^, that in our case means allocation of probability mass to negative values, by considering distributions with a positive support or by applying convenient transformations to distributions defined on the whole real line. We accomplish the latter task by truncation or using a log-transformation. Thirdly, while the aforementioned works are concerned with the whole amount of claims, we fit the competing models splitting the data by gender to see whether relevant differences exist. Finally, we test whether gender makes differences in all (or some of) the parameters of the model used to describe the distribution of claims, and we introduce a bootstrap-based parametric test to see whether significant statistical differences exist between the value at risk (VaR) predicted by the various fitted models for females and males.

### Regression modelling

With the second research question we want to assess whether gender has an effect on the magnitude of the claims, controlling for other available covariates. However, traditional regression techniques are problematic when dealing with actuarial data. Rousseeuw et al.^31^ point out that in many applications (such as insurance data), outliers have relevant effects on the estimates. Traditional ordinary least squares (OLS) regression does not satisfy the requisite of robustness, because it is sensitive to outliers. Indeed, in the OLS method the underlying distribution is Gaussian^32^ whereas insurance data, as discussed in “Distribution modelling”, depart severely from a Gaussian distribution. For these reasons, traditional OLS cannot be used for our purpose. Among the many alternative models that can solve these problems, quantile regression gained the favour of many analysts thanks to the fact that quantiles, such as the median, are less sensitive to outliers; moreover, quantile regression models are distribution-free^33^. However, Rigby et al.^34^ note that “*quantile (and expectile) regressions are less reliable in the extreme tails of the distribution because of sparsity of data points*”. For this reason, the authors consider an alternative procedure for modelling the tail of a distribution under a regression perspective, which is used in the present work (see “Regression results”). From the point of view of an insurer, knowing the behaviour of the data in the tail of the distribution is fundamental to prevent and assess adequately the largest losses. Then, we also explore the relationship between extreme losses and gender.

## Data

We worked with two important insurance datasets. The choice of these datasets descends from the need of having enough covariates and a variable for gender. It is important to note that while these are large and reliable datasets, they are country-specific and therefore our results are difficult to generalize. An in-depth discussion of this issue is provided in “Potential limitations”.

### The automobile bodily injury claims (AutoBi) dataset

The first dataset is freely available in the R package *insuranceData* and is called “Automobile Bodily Injury Claims” (AutoBi). This dataset derives from a 2002 survey conducted by the Insurance Research Council (IRC), a division of the American Institute for Chartered Property Casualty Underwriters and the Insurance Institute of America. The survey asked participating companies to report claims closed with payment during a designated 2-week period. The sample available in the package is made by 1340 bodily injury liability claims.

The variable of our interest is the claimant’s total economic loss (abbreviated as *Loss*) in thousands of dollars from a single state. Furthermore, thanks to the variable *Clmsex*, i.e. the claimant’s gender, we were able to subset the original data dividing the losses for men and women. The split of the data causes the loss of some observations since the claimant’s sex is not available for all the reported losses. The variable *Loss* is also used in the regression model as dependent variable; however, for the description of the model and the included covariates we invite the reader to look at “AutoBi”. This dataset is also used, among the others, by Frees^35^ in his book.

### The automobile claim datasets in Australia (ausprivauto0405)

The second dataset is freely available in the R package *CASdatasets* and is named “Automobile claim datasets in Australia”. Specifically, we use the dataset ausprivauto0405, made of 67,856 observations, which represent 1-year vehicle insurance policies taken out in 2004 or 2005 in Australia. Among the available policies, 4624 have at least one claim, the rest of the data are all zeros. All the losses are expressed in Australian dollars, but for scaling purposes, we rescaled the data to work with hundreds of dollars. In this case there are no missing observations. The rescaled variable *ClaimAmount* is also the dependent variable for the regression model, but all the information regarding the model are provided in “ausprivauto0405”. This dataset is also used, among the others, by De Jong and Heller^36^ in their book. It is important to note that given the presence of many zeros, all the models considered for this dataset have been zero adjusted, which means including a probability mass at 0^37^. In this way we have two different views for the phenomenon: the first dataset is focused only on losses, whereas the second one considers also policy holders without reported losses, in this way accounting for the possibility that car accidents can be more frequent depending on driver’s gender.

## Methodology

As already detailed in “Literature review”, we evaluate the variable of interest, namely the *Loss* variable (denoted by *Y*), from the point of view of its distribution (“Distribution modelling”) and as a function of some covariates of interest ${\varvec{Z}}$ (“Regression modelling”), giving particular attention to the *Gender* variable. For uniformity sake, we handle both these research objectives under a model-based paradigm which uses the very flexible family of generalized additive models for location, scale and shape (GAMLSS), proposed by Rigby and Stasinopoulos^38^ to overcome some of the limitations associated with the generalized linear models (GLMs)—such as, for example, the exponential family distribution assumption for the response variable—and generalized additive models (GAMs). In the GAMLSS methodology, the systematic part of the model is expanded to allow equations not only for the mean, but also for the other parameters (scale and shape) of the distribution of the response variable.

### The GAMLSS regression framework

A GAMLSS model can be expressed as

1 $$\begin{aligned}&Y\overset{\text {ind}}{\sim }{\mathcal {D}}(\mu ,\sigma ,\nu ,\tau )&\nonumber \\&{\left\{ \begin{array}{ll} \eta _1=g_1(\mu )=\varvec{\beta }_1'\varvec{Z}_1+s_{11}({z}_{11})+\cdots +s_{1J_1}({z}_{1J_1})\\ \eta _2=g_2(\sigma )=\varvec{\beta }_2'\varvec{Z}_2+s_{21}({z}_{21})+\cdots +s_{2J_2}({z}_{2J_2})\\ \eta _3=g_3(\nu )=\varvec{\beta }_3'\varvec{Z}_3+s_{31}({z}_{31})+\cdots +s_{3J_3}({z}_{3J_3})\\ \eta _4=g_4(\tau )=\varvec{\beta }_4'\varvec{Z}_4+s_{41}({z}_{41})+\cdots +s_{4J_4}({z}_{4J_4})\\ \end{array}\right. }&\end{aligned}$$

where ${\mathcal {D}}(\mu ,\sigma ,\nu ,\tau )$ is a four-parameter distribution (but it can have less or more parameters), with $\mu$ and $\sigma$ usually characterizing location and scale, respectively, and with $\nu$ and $\tau$ known as shape parameters (i.e., skewness and kurtosis). We denote with $i=1,\ldots ,4$ the generic *i*th equation in the system, $\eta _i$ is a predictor of the parameter (one for each of the four parameters), $g_i(\cdot )$ is a function to model the parameter of the distribution (in the empirical part of the paper we use the default functions of the commands gamlss, gamlssML, and gamlssZadj), $\varvec{Z}_i$ is a vector of covariates, $\varvec{\beta }_i$ is the coefficient vector, and $s_{ij}(\cdot )$ is a nonparametric smoothing function applied to the covariate $\varvec{z}_{ij}$, $j=1,\ldots ,J$, with *J* denoting the number of covariates. The smoothing terms $s_{ij}(\cdot )$ introduce nonlinearities in the model, and are unspecified functions estimated using a scatterplot smoother, in an iterative procedure called the *local scoring algorithm*^39^.

The form of ${\mathcal {D}}(\mu ,\sigma ,\nu ,\tau )$ is general and only implies that the distribution should be in parametric form; it can be any distribution (including highly skew and kurtotic continuous and discrete distributions) and it can model heterogeneity (e.g., cases where the scale or shape of the distribution of the response variable changes with explanatory variables). All the distributions defined on $(0,\infty )$ can be zero adjusted to $[0,\infty )$ by including a probability mass at zero using the *gamlss.inf* package^40^. The resulting new distribution can then have up to five parameters, the four parameters of the original distribution defined on $(0,\infty )$ plus a parameter $\xi _0=p_0=P(Y=0)\in \left( 0,1\right)$ that represents the probability mass at 0. Computationally, the function gen.Zadj() creates a mixed (continuous-discrete) probability density function (pdf) given by

2 $$\begin{aligned} f(y|\mu ,\sigma ,\nu ,\tau ,\xi _0)= {\left\{ \begin{array}{ll} \xi _0 &{} \hbox { if}\ y = 0, \\ (1-\xi _0)f(y|\mu ,\sigma ,\nu ,\tau ) &{} \hbox { if}\ y > 0. \end{array}\right. } \end{aligned}$$

where $f(y|\mu ,\sigma ,\nu ,\tau )$ denotes the pdf on $(0,\infty )$.

### How the research objectives of the paper are handled

Firstly, we look for the best model for the loss distribution (see “Distribution modelling” for related literature) and we investigate whether *Gender* makes differences in some aspects of the distribution such as, for example, location or scale. We handle this first objective by regressing all the parameters $\mu$, $\sigma$, $\nu$ and $\tau$ of ${\mathcal {D}}(\mu ,\sigma ,\nu ,\tau )$ on *Gender*, i.e. on only one covariate ($Z_1=Z_2=Z_3=Z_4=Z$) in (1). Thus, in case of differences due to gender in the loss distribution, that we can identify by looking at the significance of the coefficients $\beta _1$, $\beta _2$, $\beta _3$ and $\beta _4$ in (1), we have the advantage to detect the aspect(s) (location, scale and/or shape) affected by this variable.

We try several models for the loss distribution not only to have a large set of models within which to look for the best one, but also to make the evaluation of gender differences more robust with respect to a wrong model specification. Thanks to the package *gamlss* and its extensions^41,42^, we consider both classical distributions already defined on $(0,\infty )$ and new distributions on $(0,\infty )$. These new distributions are created from those with support $(-\infty ,\infty )$, using the inverse log (i.e. the exponential) transformation through the function gen.Family() with argument type=“log”, and by truncation using the function gen.trun()^42^. In detail, we consider the following 30 parametric models: Box-Cox Cole and Green, Box-Cox Power Exponential, Box-Cox *t*, Burr, Dagum (Burr III), Exponential, Gamma, Generalized Beta type 2, Generalized Gamma, Generalized Inverse Gaussian, Generalized Pareto, Inverse Gamma, Inverse Gaussian, Log-Gumbel, Log-Johnson’s SU, Log-Logistic, Log-Normal, Log-Power Exponential, Log-Skew Normal Type 2, Log-Skew *t* Type 5^43^, Log-*t* Family, Pareto Type 2, Truncated Exponential Gaussian, Truncated Johnson’s SU, Truncated Logistic, Truncated Normal, Truncated Power Exponential, Truncated Skew *t* Type 5^43^, Truncated *t* Family, Weibull.

The distributions were fitted via the maximum likelihood (ML) approach. It must be noted that, for the ausprivauto0405 dataset, we did not implement all the distributions because of computational problems related with the zero adjusted routine^44^. However, considering that we use a large number of distributions, it should not be a great loss to exclude these models from the analysis. Once the regression models are fitted, we rank them via the Akaike information criterion (AIC^45^) and by the Bayesian information criterion (BIC^46^), which represent the most popular criteria in the actuarial literature^16,18,27,28^.

Secondly, as concerns the objective of assessing the impact of *Gender* on *Loss*, controlling for other covariates, we always use the GAMLSS regression framework to model the whole distribution and its tail. The research question in this case pertains to whether female claimants generate higher losses for insurers such that the application of higher rates can be supported by a “fair justification”^11^. The use of heavy-tailed distributions overcomes the problem of extreme values in actuarial datasets. Nonetheless, knowing how gender impacts the mean or one of the other parameters of the losses distribution is less interesting for insurers than knowing the impact of gender on the tail of the distribution, where the highest losses are placed. To study this portion of the data, without recurring to nonparametric methods like the less reliable quantile regression^34^ or more complex approaches like entropic/symbolic methods^47^, we use a procedure that can be found in “Regression results” of the present paper^34,48^.

### Comparing the tail behaviour

Comparing the female and male distributions in their tails is important information for insurers because of its relation to VaR. In detail, we define a parametric (model-based) bootstrap test that can be schematized as follows.

1. Compute the sample values at risk, $\text {VaR}^F_\alpha$ and $\text {VaR}^M_\alpha$, separately for females and males, but at the same probability level $\alpha$, and compute the test statistic $\text {AD}_{\text {obs}}=\left| \text {VaR}^F_\alpha -\text {VaR}^M_\alpha \right|$.
2. Fit the GAMLSS model of interest—${\mathcal {D}}(\mu ,\sigma ,\nu ,\tau )$ or ${\mathcal {D}}(\mu ,\sigma ,\nu ,\tau ,{\xi }_0)$, depending on the available data—to the whole data of size $n=n_{\text {F}}+n_{\text {M}}$, where $n_{\text {F}}$ and $n_{\text {M}}$ are the sample sizes for females and males, respectively.
3. For $$r=1,\ldots ,B$$:
  - generate two samples of sizes $n_{\text {F}}$ and $n_{\text {M}}$ from the model fitted at step 2;
  - compute the AD statistic, say $\text {AD}_r$, on the generated samples.
4. Under $H_0$ (VaRs for males and females are statistically non-different), $\text {AD}_1,\ldots ,\text {AD}_B$ are equally likely and the *p* value of the testing procedure can be computed as
  $$\begin{aligned} p_{\text {Boot}}=1-F_{\text {Boot}}\left( \text {AD}_{\text {obs}}\right) , \end{aligned}$$
  where $F_{\text {Boot}}\left( \cdot \right)$ is the (stepwise) cumulative distribution function of $\text {AD}_1,\ldots ,\text {AD}_B$^49^.

In real data analyses, whose results are described in “Distribution fitting results”–“Regression results”, we consider a sufficiently large number of bootstrap replicates ($B=1000$); moreover, as usual in the insurance practice/literature, we consider the probability levels 0.95 and 0.99.

## Distribution fitting results

### AutoBi data

We start with the AutoBi data described in “The automobile bodily injury claims (AutoBi) dataset”. Supplementary figures C.1–C.3 in Appendix C (online) show histograms and normal Q–Q plots for the total amount of losses (Supplementary figure C.1), for the losses reported by female claimants (Supplementary figure C.2), and for the losses reported by male claimants (Supplementary figure C.3). On the histograms we superimpose also a kernel density estimate (the red line) to give an idea on how the density of the observed data should look like. The horizontal axis of the histograms in Supplementary figures C.1–C.2 is restricted to 250 for the sake of readability.

From the Q–Q plots we see that the distribution of losses for both females and males cannot be approximated by a Gaussian distribution (which is quite obvious); furthermore, the underlying distributions appear to be right skewed and heavy-tailed, as we expected. From all the histograms we confirm another recurrent feature of insurance loss data: the presence of a large amount of small losses and a lower number of high losses^16,18^. However, it should be noted that the maximum loss is registered for female claimants (1067.70), whereas the maximum for male claimants is much smaller (222.41). The kernel density estimate in the three cases seems to suggest a similar distribution, highly right-skewed and highly peaked. Further detailed information on the differences among the data can be obtained looking at the descriptive statistics in Table 1. The mean loss is higher for females than males; however, looking at the median (less sensitive to extreme values) we see that there are no remarkable differences. Nonetheless, the variability (and then the risk) is much higher for females, as evidenced by the range and by the standard deviation. The females data are also more skewed and exhibit a more pronounced leptokurtosis. The VaR shows that an insurer should expect (with confidence at 99%) higher losses for male policy holders.

**Table 1:** Automobile bodily injury claims dataset: descriptive statistics of loss data.

Automobile bodily injury claims	Total claims	Females	Males
No. observations	1340	742	586
Mean	5.95	6.21	5.65
Median	2.33	2.23	2.37
1st quartile	0.64	0.69	0.63
3rd quartile	4.00	4.03	3.90
St. Dev.	33.14	41.78	17.35
Skewness	25.66	22.63	8.29
Excess kurtosis	790.48	561.34	81.72
Minimum	0.01	0.01	0.03
Maximum	1067.70	1067.70	222.41
Range	1067.69	1067.69	222.38
99% quantile (VaR)	67.82	57.95	75.37
Tail VaR	202.91	242.04	147.32

Supplementary Tables A.1–A.3 in Appendix A (online) show the results of the distribution fitting. The results can be summarized as follows. First, we see that among the best models we have the Box-Cox *t* (selected by both the AIC and BIC as the best model for the total losses and females’ losses), the Truncated *t* and the Truncated Skew-*t*. Similar results are obtained for the female and male claimants, with a good performance of the Log-Johnson’s SU model, whereas also the Generalized Pareto and the Log-Power Exponential are competitive models. Second, we do not observe drastic differences in the selection of models for females and males. Finally, we see that distributions often neglected in applied works, such as the generalized Pareto or the log-Johnson’s SU, represent good alternatives to traditional models, whereas the variants of the normal distribution perform poorly for these data.

In order to check whether gender may explain differences in the loss distribution, we ran a GAMLSS regression for each model as described in the first part of “How the research objectives of the paper are handled”. The results are reported in Table 2. The coefficient of gender was significant only for few distributions parameters and for an exiguous number of distributions. This is a strong evidence against the fact that the loss distribution is affected by gender, regardless of the considered parametric model.

**Table 2:** AutoBi: simple regression on gender for all the parameters of the considered models.

Model	$\mu$	$\sigma$	$\nu$	$\tau$
Box-Cox *t*	0.0399 (0.1780)	− 0.0416 (0.0877)	0.0636 (0.0501)	− 0.0690 (0.4849)
Exponential	− 0.1845 (0.0611)*	.	.	.
Gamma	− 0.1845 (0.080)*	− 0.0587 (0.0362)	.	.
Generalized Beta type 2	0.1187 (0.0903)	− 0.1584 (1.2784)	0.1106 (1.2924)	0.2847 (1.2884)
Generalized Gamma	0.1432 (0.1257)	− 0.0308 (0.0438)	0.1884 (0.0900)	.
Generalized inverse Gaussian	− 0.1863 (0.1021)	0.1810 (0.0744)*	0.4288 (0.0841)*	.
Generalized Pareto	0.2429 (0.2333)	0.2062 (0.1683)	.	.
Inverse Gamma	− 0.5613 (0.0974)*	0.1233 (0.0358)*	.	.
Inverse Gaussian	− 0.1845 (0.1837)	0.2107 (0.0432)*	.	.
Log-Gumbel	− 0.0672 (0.0942)	− 0.0692 (0.0417)	.	.
Log-Johnson’s SU	− 0.0365 (0.0955)	− 0.0090 (0.1002)	− 0.1783 (0.1732)	0.0275 (0.2177)
Log-Logistic	0.0097 (0.0841)	− 0.0108 (0.0515)	.	.
Log-Normal	− 0.0423 (0.0882)	− 0.0003 (0.0432)	.	.
Log-Power Exponential	− 0.0668 (0.0919)	− 0.0159 (0.0606)	0.1022 (0.1553)	.
Log-Skew Normal Type 2	0.1793 (0.1582)	− 0.0315 (0.0445)	− 0.1108 (0.0665)	.
Log-Skew *t* Type 5	0.2240 (0.2701)	− 0.0792 (0.1009)	− 0.0805 (0.0721)	0.0674 (0.4411)
Log-*t* Family	0.0134 (0.0895)	− 0.0575 (0.0844)	− 0.3346 (0.5529)	.
Pareto Type 2	0.2432 (0.2334)	− 0.2064 (0.1683)	.	.
Truncated Johnson’s SU	− 2.5187 (9.1011)	2.6975 (1.3171)*	− 0.2589 (0.5035)	0.4189 (0.2784)
Truncated logistic	0.8297 (0.5796)	− 0.1747 (0.0511)*	.	.
Truncated Skew *t* Type 5	− 0.4246 (1.0677)	0.0887 (0.2486)	0.1390 (3.6180)	− 0.1869 (0.4277)
Truncated *t* family	− 0.4871 (0.9336)	0.0708 (0.1025)	0.1892 (0.1649)	.
Weibull	− 0.0671 (0.0942)	0.0692 (0.0417)	.	.

For some distribution it was not possible to run the regression. Standard errors are given in parentheses. *Stands for 5% significance.

Supplemntary tables A.4–A.6 in Appendix A (online) show the VaR at 95% and 99% (computed numerically) for the three typologies of data for each of the selected models. We compared these results with the observed VaRs. In this case the ranking is very different because is based on the fact that the best distribution is the one that minimises the absolute distance from the empirical VaR. Summarily, we note that the results are very different if we consider a different confidence level. Furthermore, the results for the males in this case seem to differ from the results for the females. This is reasonable since extreme values are placed in the tail of the distribution. To test if these tail differences are statistically significant, we performed the parametric bootstrap test illustrated in “Comparing the tail behaviour”; the results are reported in the left part of Table 3. For many models the differences resulted statistically significant; therefore, we should conclude that for these models the tail behaviour differs by gender. This does not necessarily imply that female claimants are riskier than male claimants, it simply means that VaRs are different.

**Table 3:** *p*-values of the parametric bootstrap tests for the hypothesis that the predicted VaRs by the models for males and females are statistically non-different.

AutoBi	VaR 95%	VaR 99%	ausprivauto0405	VaR 95%	VaR 99%
Box-Cox, Cole and Green	0.098	0.133			
Box-Cox power exponential	0.055	0.165			
Box-Cox *t*	0.058	0.309	ZA Box-Cox *t*	0.825	0.184
Burr	0.000	0.003			
Dagum (Burr III)	0.956	0.988			
Exponential	0.003	0.000	ZA exponential	0.934	0.017
Gamma	0.028	0.001	ZA gamma	0.915	0.046
Generalized beta type 2	0.128	0.255			
Generalized gamma	0.085	0.127	ZA generalized gamma	0.729	0.390
Generalized inverse Gaussian	0.166	0.075	ZA generalized inverse Gaussian	0.840	0.099
Generalized Pareto	0.835	0.954	ZA generalized Pareto	0.894	0.081
Inverse gamma	0.910	0.996			
Inverse Gaussian	0.430	0.493	ZA inverse Gaussian	0.892	0.110
Log-Gumbel	0.036	0.010	ZA Log-Gumbel	0.918	0.055
Log-Johnson’s SU	0.052	0.344	ZA Log-Johnson’s SU	0.808	0.197
Log-Logistic	0.280	0.578	ZA Log-logistic	0.888	0.035
Log-Normal	0.144	0.256	ZA Log-normal	0.867	0.041
Log-Power Exponential	0.355	0.618	ZA Log-Power Exponential	0.899	0.081
Log-Skew normal Type 2	0.058	0.090	ZA Log-skew normal Type 2	0.821	0.158
Log-Skew *t* Type 5	0.057	0.295	ZA Log-skew *t* Type 5	0.839	0.139
Log-*t* family	0.226	0.547	ZA Log-*t* family	0.882	0.041
Pareto Type 2	0.058	0.170			
Truncated exponential Gaussian	0.000	0.000	ZA truncated exponential Gaussian	0.935	0.017
Truncated Johnson’s SU	0.055	0.180			
Truncated logistic	0.000	0.000	ZA truncated logistic	0.933	0.016
Truncated normal	0.008	0.000	ZA truncated normal	0.939	0.016
Truncated power exponential	0.056	0.139	ZA truncated power exponential	0.417	0.310
Truncated skew *t* Type 5	0.998	0.995	ZA truncated skew *t* Type 5	0.761	0.305
Truncated *t* family	0.055	0.259	ZA truncated *t* family	0.884	0.131
Weibull	0.044	0.014	ZA Weibull	0.894	0.060

95% and 99% levels are considered. ZA stands for zero-adjusted.

### ausprivauto0405 data

We now analyse the distribution fitting results for the ausprivauto0405 data. Supplementary figures C.4–C.6 in Appendix C (online) show histograms and normal Q–Q plots for the total amount of losses (Supplementary figure C.4), for the losses reported by female claimants (Supplementary figure C.5) and for the losses reported by male claimants (Supplementary figure C.6). We remember that for scaling purposes the variable *ClaimAmount* is expressed in hundreds of dollars; furthermore, since we are considering only reported losses, we have excluded for the moment the zeros. In this case there was no need to restrict the horizontal axis of the histograms. The analysis of the histograms and of the normal Q–Q plots confirm the findings observed in the first dataset and characterising the majority of claims data: non-normality deriving from severe right skewness and heavy-tailed distributions, and the fact that the majority of the observations are concentrated in the first bins of the histograms. The analysis of the plots including also the zeros is redundant.

Table 4 shows the descriptive statistics for the ausprivauto0405 data (zeros excluded), whereas Table 5 shows the same statistics including also the zeros. We note that with respect to the other dataset, the losses for males are higher, on average and median, and more variable than the females. The females’ loss distribution is slightly more peaked but less skewed, whereas the males’ distribution including also the zeros shows higher kurtosis and skeweness. The VaR shows that an insurer should expect (with confidence at 99%) higher losses for male policy holders.

**Table 4:** ausprivauto0405: descriptive statistics of loss data excluding the zeros.

	Total claims	Females	Males
No. observations	4624	2648	1976
Mean	20.14	18.54	22.30
Median	7.62	7.43	8.00
1st quartile	3.54	3.54	3.54
3rd quartile	20.91	20.26	22.53
St. Dev.	35.49	30.19	41.45
Skewness	5.04	4.62	4.94
Excess kurtosis	40.21	37.06	35.57
Minimum	2.00	2.00	2.00
Maximum	559.22	472.97	559.22
Range	557.22	470.97	557.22
99% quantile (VaR)	179.37	143.12	210.61
Tail VaR	251.42	198.64	294.91

**Table 5:** ausprivauto0405: descriptive statistics of claims data including also the zeros.

	Total claims	Females	Males
No. observations	67856	38603	29253
No. of zeros	63232	35955	27277
Mean	1.37	1.27	1.51
Median	0.00	0.00	0.00
1st quartile	0.00	0.00	0.00
3rd quartile	0.00	0.00	0.00
St. Dev.	10.56	9.19	12.14
Skewness	17.50	15.85	17.66
Excess kurtosis	479.89	417.74	456.52
Minimum	0.00	0.00	0.00
Maximum	559.22	472.97	559.22
Range	559.22	472.97	559.22
99% quantile (VaR)	36.25	34.32	38.06
Tail VaR	82.99	74.16	94.37

Supplementary Tables B.1–B.3 in Appendix B (online) show the results of the distribution fitting. The ZA Generalized Gamma was selected as the best model by both the AIC and BIC for the total claims, and both the females and males claims. The ZA Log-Skew Normal, the ZA Log-Johnson’s SU and the ZA Generalized Inverse Gaussian were competitive models for all the three groups of data. Table 6 shows that, for this dataset, gender seems to play a role in explaining differences in the location parameter, and for some distributions also the dispersion parameter. As for the AutoBi data there is weak evidence that gender could explain the shape of the distribution.

**Table 6:** ausprivauto0405: simple regression on gender for all the parameters of the considered models.

Model	$\mu$	$\sigma$	$\nu$	$\tau$
ZA Box-Cox *t*	− 9.2930 (3.6930)*	0.0650 (0.0210)*	− 0.0264 (0.0010)*	12.0604 (0.7143)*
ZA exponential	− 0.1847 (0.0297)*	.	.	.
ZA gamma	− 0.1847 (0.0345)*	− 0.0535 (0.0180)*	.	.
ZA generalized gamma	− 0.0036 (0.0057)	− 0.0337 (0.0264)	− 0.7137 (2.5784)	.
ZA generalized inverse Gaussian	− 0.1845 (0.0642)*	− 0.1329 (0.0726)	0.0257 (0.0687)	.
ZA generalized Pareto	0.1460 (0.1216)	0.1799 (0.0868)*	.	.
ZA inverse Gaussian	− 0.1846 (0.0503)*	0.0065 (0.0210)	.	.
ZA Log-Gumbel	− 0.1173 (0.0405)*	− 0.0645 (0.0211)*	.	.
ZA Log-Johnson’s SU	− 0.0845 (0.0408)*	− 0.0239 (0.0478)	− 5.331 (240.977)	0.0178 (0.0877)
ZA Log-logistic	− 0.0752 (0.0369)*	− 0.0324 (0.0243)	.	.
ZA Log-Normal	− 0.0875 (0.0355)*	− 0.0412 (0.0210)	.	.
ZA Log-Power Exponential	− 0.0479 (0.0439)	− 0.0397 (0.0170)*	0.2051 (0.0875)*	.
ZA Log-skew normal Type 2	0.1530 (0.0276)*	0.6424 (0.0241)*	− 0.7023 (0.0249)*	.
ZA Log-skew *t* Type 5	− 0.1206 (0.4222)	0.0459 (0.7903)	− 0.0208 (0.0441)	− 0.0141 (0.8297)
ZA Log-*t* Family	− 0.0875 (0.0355)*	− 0.0412 (0.0210)	− 6.661e−26 (2.973e−07)	.
ZA truncated logistic	30.01 (40.02)	− 0.1847 (0.0297)*	.	.
ZA truncated skew *t* Type 5	0.1598 (0.1543)	− 0.2641 (1.2847)	0.0106 (0.0465)	− 0.1152 (1.2847)
ZA truncated *t* family	− 0.7710 (0.7212)	0.0592 (0.0966)	0.1701 (0.1007)	.
ZA Weibull	− 0.1172 (0.0404)*	0.0645 (0.0210)*	.	.

For some distribution it was not possible to run the regression. Standard errors are given in parentheses. The estimate for $\xi _0$ is − 0.0165 (s.e. = 0.0308) for all models. *Stands for 5% significance.

Supplementary Tables B.4–B.6 in Appendix B (online) show the estimated VaR values at 95% and 99% using the ZA parametric models. We can say that ZA Truncated Power Exponential, ZA Generalized Pareto and ZA Log-Skew Normal are good models to describe the tail behaviour of these data. As in the previous dataset there are differences between the ranks obtained using the two different levels. However, in this case the VaR bootstrap tests highlight that there are no significant differences in the tail of the distribution of male and female claimants when we consider a level of 95%, whereas significant differences emerge for a level of 99% (see Table 3).

## Regression results

In this section we tackle the second research question of the paper, i.e. whether gender affects the claims distribution controlling for other available covariates. We fit regression models on the whole dataset and on the right tail of the data. The former approach is useful to quantify the effect of gender on the conditional location, scale and shape of the losses, the latter to quantify the effect of gender on the largest claims. For insurance companies this information is of relevant importance because it influences the solvency of the company and its policies. The GAMLSS framework consents to exploit the results of the distribution fitting in order to use the best model as underlying distribution.

The choice of functions $g_i(\cdot )$, $i=1,\ldots ,4$, to model the parameters of the considered models (refer to “The GAMLSS regression framework”) is limited to those available in the *gamlss* package. To model the tail of the data we used a different approach^34,48^. These are synthetically the steps followed.

1. We fitted a $\alpha$ (95% and 99%) smooth quantile curve for LOSS (or ClaimAmount) against the explanatory variables using the R package *cobs* with automatic smoothing parameter selection.
2. We selected the cases above the $\alpha$ quantile curve to work only with the tail of data.
3. We fitted a suitable GAMLSS truncated distribution to the tail data with the fitted $\alpha$ quantile as truncation parameter. Since fitting via regression all the distributions is computationally prohibitive, the choice of an adequate distribution is determined using the best models obtained in “Distribution fitting results”. For the whole dataset we used the best model on the total claims distribution, while for the tail of data we used the best model as suggested by the VaR difference between the empirical VaR and the theoretical VaR. For the asprivauto0405 dataset we used GAMLSS zero-adjusted distributions.
4. We fitted regression models to assess the magnitude of the gender coefficient on the distribution of claims using, for the tail of data, the truncated distribution as determined in step 3.

### AutoBi

The AutoBi dataset contains the following explanatory variables:

- *Attorney*: whether the claimant is represented by an attorney.
- *Clmsex*: claimant’s gender.
- *Marital*: claimant’s marital status (= 1 if married, =2 if single, = 3 if widowed, and = 4 if divorced/separated).
- *Clminsur*: whether or not the driver of the claimant’s vehicle was uninsured.
- *Seabelt*: whether or not the claimant was wearing a seatbelt.
- *Clmage*: claimant’s age.

As before, the dependent variable of the regression model is *Loss*, the claimant’s total economic loss (in thousands of dollars). In order to perform the regression model, we create dummy variables for *Attorney* (1 if yes), for *Clmsex* (1 if female), for each marital status, for *Clminsur* (1 if yes) and for *Seatbelt* (1 if yes). To avoid the *dummy variables trap* we exclude from the regression the dummy relative to divorced/separated, which becomes the benchmark category. Due to the presence of missing observations we use listwise deletion to eliminate the rows with missing information, therefore, the final dimension of the dataset in terms of rows is 1091.

**Figure 1:**
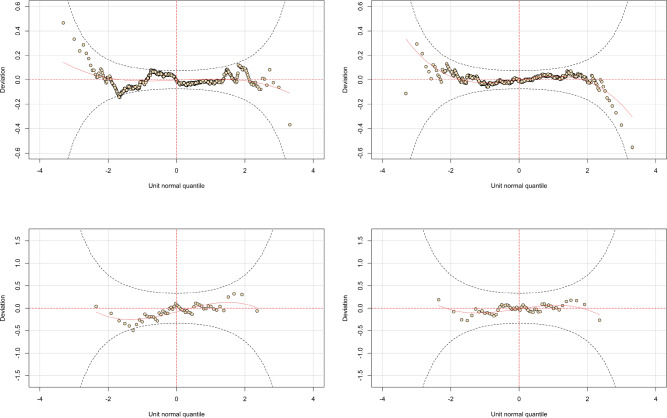
Wormplots of models I–IV (Tables 7, 8) for the AutoBi data. Upper panels: model I on the left, model II on the right. Lower panels: model III on the left, model IV on the right.

Tables 8 and 9 show the result of the GAMLSS regressions. We could not fit the best model for the 99% quantile because the cases above it are too few to fit a suitable regression model. Figure 1 shows the wormplots for the AutoBi data. We used also other graphical tools for diagnostics and we estimated many models but we omit them from this paper for the sake of synthesis. The interested reader can contact the corresponding author for further elaborations.

**Table 7:** Results of the GAMLSS regression on the AutoBi data.

	$\mu$ coefficients	$\sigma$ coefficients	$\nu$ coefficients	$\tau$ coefficients
I: whole dataset				
Intercept	2.5925 (1.2747)**	− 0.0001 (0.0423)	0.0720 (0.0270)***	1.7066 (0.1969)***
Attorney	2.3129 (0.1760)***			
Clmsex	0.1855 (0.0859)**			
Clminsur	0.0139 (0.1891)			
Seatbelt	− 1.8296 (1.0807)*			
Clmage	0.0216 (0.0037)***			
Married	− 0.3433 (0.6746)			
Single	− 0.5406 (0.6771)			
Widowed	− 1.2191 (0.9152)			
Distribution	Box-Cox *t*			
No. observations	1091			
AIC	4893.469			
BIC	4953.408			

	$\mu$ coefficients	$\sigma$ coefficients	$\nu$ coefficients	$\tau$ coefficients
II: whole dataset				
Intercept	0.5321 (0.2248)**	0.7718 (0.2710)***	1.9517 (0.1255)***	13.9009 (0.5263)***
Attorney	3.0709 (0.1521)***	− 0.5594 (0.0639)***	− 1.0346 (0.0501)***	− 15.0164 (0.3038)***
Clmsex	0.1991 (0.0815)**	− 0.0161 (0.0557)	0.0637 (0.0456)	0.0621 (0.1237)
Clminsur	− 0.1176 (0.1700)	0.0611 (0.0983)	− 0.1273 (0.0792)	0.1921 (0.2046)
Seatbelt	− 0.0534 (0.1744)	− 0.6794 (0.2576)***	− 1.8099 (0.1032)***	0.5558 (0.3414)
Clmage	0.0192 (0.0034)***	0.0035 (0.0019)*	0.0004 (0.0015)	0.0248 (0.0051)***
Married				
Single	− 0.2136 (0.1196)*	0.0158 (0.0647)	− 0.1135 (0.0532)**	− 0.2807 (0.1591)*
Widowed	− 1.4037 (0.3725)***	0.7564 (0.2249)***	0.3235 (0.1068)***	1.5982 (0.3568)***
Distribution	Box-Cox *t*			
No. observations	1091			
AIC	4774.886			
BIC	4934.722			

Standard errors are given in parentheses. Model I and II assume as underlying distribution the best distribution for total claims (online Supplementary table A.1, Appendix A) as univocally determined by AIC and BIC. *indicates 10% significance, **indicates 5% significance, ***indicates 1% significance.

**Table 8:** Results of the GAMLSS regression on the AutoBi dataset for the tail of data (cases above 95% quantile).

	$\mu$ coefficients	$\sigma$ coefficients	$\nu$ coefficients	$\tau$ coefficients
III: cases above 95% quantile				
Intercept	− 47.6269 (34.7362)	2.1297 (0.7092)***	− 0.7141 (1.7241)	0.8694 (0.5841)
Attorney	31.3194 (7.0223)***			
Clmsex	− 31.3352 (5.3629)***			
Clminsur	3.2517 (9.8131)			
Seatbelt	33.1880 (30.4418)			
Clmage	0.1453 (0.0961)			
Married	− 1.7097 (21.3680)			
Single	17.7032 (10.3500)*			
Widowed				
Distribution	Truncated Skew *t* Type 5			
No. observations	54			
AIC	496,293			
BIC	518,172			

	$\mu$ coefficients	$\sigma$ coefficients	$\nu$ coefficients	$\tau$ coefficients
IV: cases above 95% quantile				
Intercept	− 45.9200 (29.4962)	2.5509 (0.8069)***	− 0.9047 (3.6915)	0.9421 (0.9583)
Attorney	30.1951 (8.9841)***			
pb(Clmsex)	− 30.1765 (6.1283)***	− 3.3941 (0.3723)***		
Clminsur	5.8439 (1.4436)***			
Seatbelt	31.5471 (25.9840)			
Clmage	0.1326 (0.1275)			
Married	1.7247 (12.4398)			
Single	17.4495 (11.4281)			
Widowed				
Distribution	Truncated Skew *t* Type 5			
No. observations	54			
AIC	490.931			
BIC	514.799			

Standard errors are given in parentheses. Model III and IV assume as underlying distribution the best distribution based on the difference between the empirical VaR and the distribution-based VaR for a 95% confidence level for total claims (online Supplementary table A.4, Appendix A). *indicates 10% significance, **indicates 5% significance, ***indicates 1% significance.

#### AutoBi: regression model on total claims

In Table 7, we report the results of two regression models. In model I we model only the equation of the parameter $\mu$ using all the data and all the explanatory variables. The best model, as suggested by the analysis performed in “AutoBi data”, is the Box-Cox *t* distribution. The coefficient of our interest is the coefficient of *Clmsex*. Female claimants are associated with a positive and significant (at 5%) increase in the insurer losses (in thousands of dollars). The fit of the model is good enough as evidenced by the wormplot of the model in Fig. 1 (upper-left panel). However, we can obtain better estimates if we model also the other parameters, i.e. the scale parameter $\sigma$ and the skeweness and kurtosis parameters $\nu$ and $\tau$. To achieve this purpose, we gone through several models estimation. These models do not exhaust all the possible cases: given the fact that we can model four equations using several explanatory variables, the number of cases is high. This happens because not only can we create many models by simply changing the set of explanatory variables among those available (all models with one variable, with two variables, with three variables, and so on) but we can test these different combinations in four different equations (one for the mean, one for the dispersion parameter, and so on). However, we tried to cover all the relevant cases for the research question of this paper. These relevant cases are all those in which it was possible to retain the gender variable (given the research question of this paper), and were considered the best (using information criteria and graphical tools such as wormplots) among those with the gender variable for which the algorithm was able to converge.

Model II represents the best model, with respect to the many models that we estimated, in terms of computational feasibility(with this term we refer to the fact that some models were not computationally feasible and/or showed excessive time complexity), AIC and BIC, and goodness of fit as exhibited by the worm plot. The wormplot (Fig. 1, upper-right panel) shows a better fit since all the points lie within the 95% confidence intervals given by the two elliptic curves. The coefficient of *Clmsex* preserved the same sign and approximately the same magnitude. On the other hand, *Clmsex* does not affect significantly the other parameters of the distribution. Finally, the significant coefficients of the other explanatory variables are economically reasonable. For example, considering the $\mu$ equation, if the claimant is represented by an attorney, the insurance company tends to pay bigger amounts; if the age of the claimant increases, also the loss for the company increases, probably because elder people suffer more physical damages in car accidents.

#### AutoBi: regression model on the tail of data

The analysis for the tail of the data is reported in Table 8. In this case the best distribution is selected according the result for the VaR estimation reported in online Supplementary table A.4. Once again, we first estimate a model (III) only for the $\mu$ equation and with all the explanatory variables (*Widowed* is dropped because on 54 cases there were not sufficient observations for this variable). The other model (IV) is again the best one in the sense specified in “AutoBi”. In model IV we include a smoother for *Clmsex* (*pb* is a smoothing additive term based on P-splines) for both the $\mu$ and $\sigma$ equations. Modeling also the other equations is not possible due to the low number of cases available in the tail of data.

These results are probably more interesting for an insurer. The coefficient of *Clmsex* is strongly significant and negative in both models. This means that female claimants entail lower losses for insurers, which means that the biggest losses are made for male claimants as confirmed by other works^9,10^. In model IV we also learn that the variable *Clmsex* has a negative effect also on the scale parameter, which means that female claimants decrease the spread in the tail of the distribution. Both the wormplots of model III and IV show a satisfactory fit (Fig. 1, respectively, lower-left and lower-right panels). Once again, the presence of an attorney is associated with the biggest losses for the company.

### ausprivauto0405

The dataset asprivauto0405 contains 9 variables. The dependent variable in our study is *ClaimAmount*, which is the sum of claim payments. In this case we do not use the term loss because the variable *ClaimAmount* contains also zeros. The explanatory variables available in the dataset are:

- *Exposure*: the number of policy years.
- *VehValue*: the vehicle value in thousand of Australian dollars.
- *VehAge*: The vehicle age group divided into 4 classes: old cars, oldest cars, young cars and youngest cars. We created a dummy variable for each category.
- *VehBody*: the vehicle body group divided into 13 classes: Bus, Convertible, Coupe, Hardtop, Hatchback, Minibus, Motorized caravan, Panel van, Roadster, Sedan, Station wagon, Truck and Utility. We created a dummy variable for each category.
- *Gender*: the gender of the policyholder. We created a dummy variable for female claimants (*Female*).
- *DrivAge*: the age of the policyholder divided into 6 classes: old people, older working people, oldest people, working people, young people and youngest people.
- *ClaimOcc*: a dummy variable that indicates occurence of a claim.
- *ClaimNb*: the number of claims.

We proceed as for the AutoBi dataset with the only difference that for this dataset we use the zero-adjusted GAMLSS framework. Also in this case we estimated several models but we report only the relevant cases for the sake of synthesis, which are, as mentioned earlier, those for which the gender variable could be retained and were selected as the best model among those for which the algorithm was able to converge.

#### ausprivauto0405: regression model on total claims

We started with the ZA Generalized Gamma (GG) as underlying distribution since it was the best one to model the total amount of claims (online Supplementary table B.1, Appendix B). Unfortunately, for this model the regression algorithm cannot reach convergence and this affects the reliability of the estimates. Given the problem of convergence, we tried the second and third best models as suggested by the analysis of Supplementary table B.1 (Appendix B, online), but for the ZA Log-Skew Normal Type 2 and the ZA Truncated Power Exponential we had also the same problem. Consequently, in order to improve the reliability of the regression model we discarded them. For the fourth best model, the ZA Log-Johnson’s SU, the algorithm converged.

**Figure 2:**
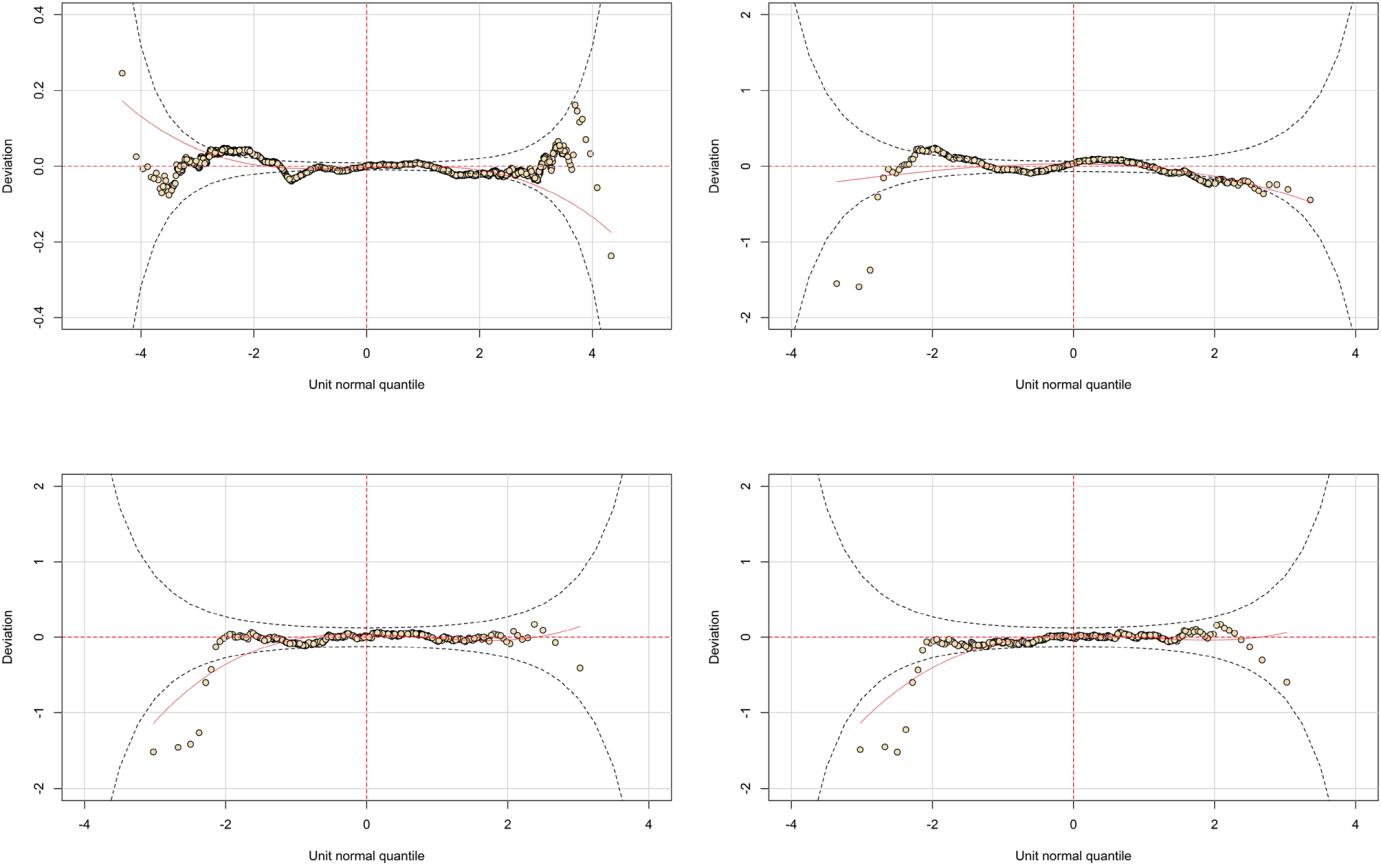
Wormplots of model V–VIII (Tables 9, 10, 11) for the ausprivauto0405 dataset. Upper panels: model V on the left, model VI on the right. Lower panels: model VII on the left, model VIII on the right.

Model V in Table 9 is the best in terms of computational feasibility, AIC, BIC, and wormplot. Nonetheless, we should warn the reader that better models could be obtained removing the variable *Female*, but this is not the purpose of this paper. Even though the coefficient of the variable *ClaimOcc* in the $\xi _0$ equation is not significant, we include it to obtain a satisfactory wormplot (Fig. 2, upper-left panel). We did not model also the equation for the $\tau$ parameter because this would have increased enormously the time complexity. Just to give an idea, Model V in Table 9 converged after 220 iterations, a model with all variables in the four parameters did not converge even after 1500 iterations (a routine of about 24 h on a computer Intel Core i7-6500U CPU with 16 GB of RAM).

The variable *Female* affects significantly both the $\mu$ and $\sigma$ parameters and the sign is negative, which means that for female claimants the location and spread of claims is lower respect to male claimants. No significant effect resulted for the coefficient of *Female* on the parameter $\nu$. We also tried a model where the variable *Female* appeared also in the $\xi _0$ equation, but the coefficient was highly non-significant. As in the AutoBi dataset we find the same effect of gender on the spread, but in this dataset, where we consider also the case of no-claims, we find that female claimants seem to be better clients for insurers also in terms of the location parameter.

**Table 9:** Results of the GAMLSS regression on the ausprivauto0405 dataset.

V: whole dataset	$\mu$ coefficients	$\sigma$ coefficients	$\nu$ coefficients	$\tau$ coefficients	$\xi _0$ coefficients
Intercept	1.8800 (0.0651)***	− 0.2998 (1.1980)	99.2859 (5.8370)***	0.5830 (0.0219)***	20.57 (70.51)
Exposure	− 0.3536 (0.0493)***	− 0.2695 (0.0420)***	− 39.9574 (4.6353)***		
VehValue	− 0.0450 (0.0105)***	− 0.0378 (0.0081)***	− 4.8144 (0.4929)***		
ClaimOcc		1.0566 (1.1977)			− 41.13 (270.11)
ClaimNb	0.7977 (0.0330)***	− 0.1487 (0.0276)***	− 29.3799 (2.6756)***		
OldCars	0.0257 (0.0291)		− 14.1199 (2.7430)***		
OldestCars			25.0335 (3.7060)***		
YoungCars	0.0053 (0.0297)	0.0171 (0.0236)			
Bus					
Convertible					
Coupe					
Hardtop					
Hatchback	− 0.1545 (0.0350)***				
Minibus			− 49.0222 (13.9223)***		
MotorizedCaravan		− 0.2267 (0.6205)			
PanelVan					
Roadster	− 0.9254 (0.2381)***	− 1.6552 (2.7983)			
Sedan	− 0.1438 (0.0311)***				
StationWagon					
Truck			47.0991 (9.2033)***		
Female	− 0.0795 (0.0253)***	− 0.0507 (0.0210)**	2.9355 (2.2786)		
Old	− 0.1505 (0.0418)***	− 0.0993 (0.0351)***	3.5518 (4.1832)		
OlderWorking	− 0.1229 (0.0363)***	− 0.0649 (0.0284 )**	18.7495 (3.4452)***		
Oldest		− 0.0273 (0.0396)	22.8866 (5.5659)***		
Working	− 0.1015 (0.0362)***	− 0.0474 (0.0282)*	25.3633 (3.1041)***		
Young	− 0.0577 (0.0400)				
Distribution	ZA Log-Johnson’s SU				
No. observations	67856				
AIC	34125.48				
BIC	34517.87				

Standard errors are given in parentheses. The model assumes as underlying distribution the ZA Log-Johnson’s SU, which is the fourth best model for total claims (online Supplementary table B.1, Appendix B) as univocally determined by AIC and BIC. *indicates 10% significance, **indicates 5% significance, ***indicates 1% significance.

#### ausprivauto0405: regression model on the tail of data

We shift now our attention to the tail of the distribution. Since now we deal with data above the 95% and 99% quantiles, we are eliminating from the analysis all the zeros and dealing only with losses. In this case the regression framework becomes again the traditional GAMLSS without any need for zero-adjustment. Moreover, including the variable *ClaimOcc* becomes redundant because in the tail there are only realised claims.

Table 10 shows the results of the best model for cases above the 95% quantile among many competing models. The choice of the Truncated Power Exponential was determined by the results obtained comparing the empirical VaR with the VaR predicted by the models (online Supplementary table B.4, Appendix B). One may notice that the analysis of VaR was conducted using ZA distributions, but this is a minor concern since the wormplot shows that the model offers a good fit for the data (Fig. 2, upper-right panel). The coefficient of *Female* is significant and positive in the $\mu$ equation, which means that claims in the tail increase for female claimants, whereas the coefficient of *Female* for the scale parameter is non-significant. We excluded the variable from the $\nu$ equation because it was non-significant and it affected severely the goodness of fit of the model.

**Table 10:** Results of the GAMLSS regression on the ausprivauto0405 dataset for the tail of data (cases above 95% quantile).

VI: cases above 95% quantile	$\mu$ coefficients	$\sigma$ coefficients	$\nu$ coefficients
Intercept	− 42.16212 (0.5185)***	3.8340 (0.1212)***	− 0.8081 (0.0762)***
Exposure	3.1479 (0.3792)***	− 1.1464 (0.1637)***	− 0.0137 (0.0826)
VehValue	7.5577 (0.0372)***	0.5357 (0.0584)***	− 0.1965 (0.0203)***
ClaimOcc			
ClaimNb	12.2939 (0.2652)***		
OldCars	6.7017 (0.2469)***		
OldestCars	7.1626 (0.0510)***		
YoungCars	11.9569 (0.1343)***		
Bus	16.6290 (0.3860)***		
Convertible			
Coupe	32.5642 (0.3108)***		
Hardtop	− 70.3922 (15.6679)***		
Hatchback	5.3664 (0.2909)***		
Minibus	4.2896 (2.5596)*		
MotorizedCaravan			
PanelVan	8.7956 (0.3004)***		
Roadster			
Sedan	3.7060 (0.3326)***		
StationWagon	3.8400 (0.3394)***		
Truck			
Female	14.1298 (0.1524)***	0.1259 (0.0907)	
Old	15.0275 (0.3314)***		0.2090 (0.1053)**
OlderWorking	15.7038 (0.4510)***		0.3637 (0.0885)***
Oldest	7.8398 (0.0978)***		− 2.1380 (0.0647)***
Working	14.1130 (0.2402)***		0.1239 (0.0827)
Young	14.6956 (0.3638)***		0.3721 (0.0924)***
Distribution	Truncated Power Exponential		
No. observations	1271		
AIC	11036.06		
BIC	11205.93		

Standard errors are given in parentheses. The model assumes as underlying distribution the best distribution (Truncated Power Exponential) based on the difference between the empirical VaR and the distribution-based VaR for a 95% confidence level for total claims (online Supplementary table B.4, Appendix B). *indicates 10% significance, **indicates 5% significance, ***indicates 1% significance.

Table 11 shows two possible models to describe the behaviour of extreme losses. Both models are good in terms of fit as highlighted by the wormplots in Fig. 2. However, model VII should be preferred in terms of AIC and BIC. In model VIII the variable *Female* was removed from the equation for the location parameter because it was non-significant. The choice of the underlying distributions is again determined by computational feasibility and the results of Supplementary table B.4 (Appendix B, online). The coefficient of the variable *Female* is negative and significant at 10% for the location parameter in model VII and for the dispersion parameter in model VIII. These results are in line with the observed tail behaviour in the AutoBi dataset (Table 8).

**Table 11:** Results of the GAMLSS regression on the ausprivauto0405 dataset for the tail of data (cases above 99% quantile).

	$\mu$ coefficients	$\sigma$ coefficients	$\nu$ coefficients
VII: cases above 99% quantile			
Intercept	3.7048 (0.2841)***	0.2710 (0.0543)***	0.6805 (0.1422)***
Exposure	− 1.4713 (0.2850)***		
VehValue	0.3191 (0.0971)***		
OldCars	− 0.6813 (0.2285)***		
OldestCars	− 0.4221 (0.2225)*		
YoungCars	− 0.4410 (0.2720)		
Hardtop	0.8007 (0.4523)*		
Truck	0.7865 (0.3801)***		
Female	− 0.3338 (0.1869)*		
Young	0.4381 (0.1998)**		
Distribution	Generalised Gamma		
No. observations	401		
AIC	3719.711		
BIC	3767.639		

	$\mu$ coefficients	$\sigma$ coefficients	$\nu$ coefficients
VIII: cases above 99% quantile			
Intercept	3.5215 (0.2256)***	0.4212 (0.1073)***	0.8286 (0.2020)***
Exposure	− 1.3589 (0.2240)***		
VehValue	0.33603 (0.0698)***	− 0.1565 (0.0687)**	
OldCars	− 0.3779 (0.1622)**		
OldestCars	− 0.3857 (0.1845)**		
Bus	− 0.6387 (0.4038)		
Hardtop	1.4903 (0.2802)***	− 0.6955 (0.3335)**	
Minibus		0.4801 (0.3583)	
StationWagon	0.5273 (0.2575)**	− 0.2607 (0.1644)	
Truck	0.7747 (0.3121)**		
Female		− 0.1612 (0.0958)*	
OlderWorking		0.2410 (0.1300)*	
Oldest		0.3590 (0.1992)*	
Working	0.4364 (0.0345)**	− 0.2297 (0.1477)	
Young	0.5494 (0.1618)***		
Distribution	Generalised Gamma		
No. observations	401		
AIC	3723.161		
BIC	3807.034		

Standard errors are given in parentheses. The underlying distribution is based on the difference between the empirical VaR and the distribution-based VaR for a 99% level for total claims (online Supplementary table B.4, Appendix B). *indicates 10% significance, **indicates 5% significance, ***indicates 1% significance.

## Potential limitations

In this section, we address a series of shortcomings that could undermine the validity of our results.

### Dataset

Finding adequate data when dealing with actuarial studies is a relevant problem. Since in most cases researchers need micro-data, these data should contain enough information, especially when one aims to run regressions. In our case a suitable dataset must report the claimant’s gender and a sufficient number of other variables to avoid endogeneity problems. Furthermore, the ideal dataset should include an high number of observations and should contain data on a relevant geographical context to draw useful policy proposals. Nonetheless, the search of these data was not painless. We think that the data used in our study are a good compromise. The AutoBi dataset allows us to study the American context, where the problem of pricing based on gender is currently relevant. Moreover, the ausprivauto0405 dataset allows us to extend the analysis to a different geographical context, including also policy holders with no claims.

One may argue that the data used are old. We think that this is not a serious problem for many reasons. It is customary in actuarial studies to work with important and established datasets. Working with reliable and significant data is more important than working with new data. Furthermore, as already mentioned, finding data is very difficult. The literature is plenty of works dealing with older but established datasets. Just to mention: the Danish Fire losses dataset contains data gathered over the period 1980–1990, yet it is still one of the most used in contemporary studies^18,27^; Fuzi et al.^33^ used private car policies in year 2001; Blostein and Miljkovic^28^ used data for the time period 1988–2001. Another relevant aspect to consider is that the distribution of claims generally presents the same statistical features over time and across countries.

We are aware of the fact that many other variables should have been added in the model, such as locations of accident, time of the accident, reason of the accident (drug, traffic rule disregard, etc.) and so on. Nonetheless, a dataset with such a detailed information, to our knowledge, is not freely accessible. The data used in this paper are among the most complete we could have found. Nevertheless, we must stress that the use of country-specific data limits the conclusions drawn from these datasets to the cases analyzed; therefore, further research using the same methodology but different data would help corroborate the results of this work. In this regard, the hope is that more insurers will make the data freely available to advance actuarial research.

### Causality

The regression models used in our analysis served to study the relationship between gender and claims; however, no causal effect can be drawn from this setup. The point is that even conceiving a study capable of assessing the existence of a causal effect is troublesome because car accidents, and hence the amount of claims, are too complex to ideate any experiment. The lack of data makes this problem even worse. Nonetheless, the study of correlations is important to investigate whether a fair justification supporting a pricing practice exists.

### External validity

One major drawback from using data of US and Australian companies is the impossibility of drawing general conclusions also for other countries. In general, a representative sample is needed to generalize the results to different countries. As one of the referees pointed out, it is reasonable to assume that our data are not representative of the many policy holders who have contracts with insurance companies. This obviously limits the application of our results to the scenarios analyzed, and their application to broader contexts depends strictly on how close one thinks our data are to a representative sample.

Despite this, our results are useful for different reasons. First, as we point out in “Introduction”, the problem of price discrimination based on gender is particularly relevant in the US. This work therefore can be used to provide statistical substance to the debate. Second, Australia and USA are two prominent markets for insurers worldwide. Third, even though driving habits are very different from country to country, countries with similar backgrounds can still use the results of our analysis. Fourth, the loss distribution is characterized by stylized facts that make the present study useful also for different data. Finally, our work can serve as a stimulus to produce further empirical evidence on this topic, providing new insights into the external validity of our results.

## Conclusions and policy implications

This paper provides several results that extend and enrich the existing literature. These results can be split into two parts. In the first part of the paper, we focus our attention on finding the best statistical model to describe the distribution of claims. The variables investigated are taken from two important R packages. The Autobi dataset allows us to work on losses, as is commonly done in the literature^16,18,27^, whereas the ausprivauto0405 includes also zeros, allowing us to adopt the zero-adjusted distribution framework. Moreover, we conduct the analysis not only on total claims but also distinguishing by gender and analysing the tail behaviour of the data.

In the first part of the paper, we learn that male and female claims can be approximated by similar distributions, for example the Truncated Skew *t* Type 5 or the Truncated *t* Family for the AutoBi dataset. Secondly, regarding the effect of gender on the parameters of the distribution, we find a significant difference for the location parameter of many distributions for the second dataset (Table 3). Finally, thanks to a parametric bootstrap test based on the difference between VaRs, we can conclude that for many distributions a significant difference exists between the tail distribution of male and female claimants. Based on this evidence, few statistical differences seem to exist between male and females. However, this just evidences that the best model to describe the data may differ by gender. Unfortunately, these results are limited by the use of the only available data we could find. Therefore, this evidence, although based on sound statistical methodology, should be supported by the analysis on additional data to be generalized.

The second part of the paper is devoted to build a GAMLSS regression model to capture the “effect” of gender on the claims reported by the insurer. In this case we conduct the analysis using all the data and the tail (cases above the 95% and 99% quantiles). It seems that for female claimants the spread of losses is lower than for male claimants. For the $\mu$ parameter the results are contrasting. For the AutoBi dataset we find evidence of a positive effect of female claimants on the location parameter when we consider all the data, whereas the effect is negative when we consider only the cases above the 95% quantile. For the ausprivauto0405 dataset we find evidence of a negative effect on location when considering all the data and on extreme losses (cases above 99%), and a positive effect when considering cases above 95%. The negative effect on the location parameter on the whole dataset is, in our opinion, a more reliable result than the positive effect for the AutoBi dataset because the inclusion of zeros accounts for the fact that females can be safer policy holders.

Nonetheless, the regression framework presents some limits. The principal limits are related to the high complexity of the computational routines and to the lack of data. We must rely on the adequacy of the control variables provided in the R packages. The strength of the empirical analysis is that the GAMLSS framework allowed us to study the phenomenon thoroughly, including also equations for the other parameters of the distribution (quite often neglected in empirical works) and weighting also the information carried by the zeros. The main limitation is the use of old, country-specific data, which reduces the scope of these results, although the analysis is robust and allows useful policy implications to be drawn for many countries.

In conclusion, our research enlightened that finding a “fair justification”^11^ for applying different rates to male and female claimants is difficult. However, female claimants seem in most of the investigated cases to decrease the location parameter for extreme losses and when zeros are included. Furthermore, in our data female claimants have a beneficial effect on the scale parameter of claims, since for females the spread of losses decreases. We do not think that these results represent incontestable statistical reasons to differentiate policy rates by gender. Indeed, if we read our results together with other works that show that female policy holders are safer than men, we do not see any clear reason to charge women with higher rates. The same argument can be made for male policy holders. The evidence collected suggests in part that men may be riskier for insurance companies in some cases, but the evidence is not strong enough to justify charging higher rates. Future research can make use of the methodology presented in this paper to see if similar results are obtained for different data. In any case, this paper offers guidance to policy makers in the countries considered on whether unisex pricing policies should be promoted.

### Supplementary Information

Supplementary Information.

## Data Availability

Data can be accessed downloading the R packages reported in the paper.
