# Supplementary material for: Women and insurance pricing policies: a gender-based analysis with GAMLSS on two actuarial datasets
Source: Sci Rep. 2024 Feb 8;14:3239. doi: 10.1038/s41598-024-52959-8 (PMC10853228; doi:10.1038/s41598-024-52959-8)
Supplement: Supplementary file 1 — Supplementary Information. [file 41598_2024_52959_MOESM1_ESM.pdf]

Appendix A. Supplementary tables associated to Section 5.1

Supplementary table A.1: `AutoBi`, total claims: log-likelihood, AIC, and BIC for 30 competing models, along with rankings based on these criteria.

Model (**total claims**)	# par.	Log-likelihood	AIC	Rank (AIC)	BIC	Rank (BIC)
Box-Cox Cole and Green	3	-3136.23	6278.46	16	6294.04	17
Box-Cox Power Exponential	4	-3229.57	6467.15	20	6487.91	20
Box-Cox t	4	-3102.73	6213.46	1	6234.23	1
Burr	3	-3124.57	6255.14	13	6270.72	13
Dagum (Burr III)	3	-3119.15	6244.30	10	6259.88	10
Exponential	1	-3699.95	7401.89	27	7407.08	27
Gamma	2	-3440.44	6884.88	25	6895.27	25
Generalized Beta type 2	4	-3139.80	6287.61	19	6308.37	19
Generalized Gamma	3	-3136.46	6278.93	17	6294.50	18
Generalized Inverse Gaussian	3	-3292.28	6590.56	23	6606.13	23
Generalized Pareto	2	-3116.53	6237.06	8	6247.44	5
Inverse Gamma	2	-3470.32	6944.63	26	6955.01	26
Inverse Gaussian	2	-3413.48	6830.95	24	6841.33	24
Log-Gumbel	2	-3265.47	6534.94	21	6545.32	21
Log-Johnson's SU	4	-3106.75	6221.51	4	6242.27	4
Log-Logistic	2	-3125.13	6254.27	12	6264.65	11
Log-Normal	2	-3139.57	6283.13	18	6293.51	16
Log-Power Exponential	3	-3122.38	6250.75	11	6266.33	12
Log-Skew Normal Type 2	3	-3129.00	6263.99	15	6279.57	15
Log-Skew t Type 5	4	-3114.34	6236.68	6	6257.45	8
Log-t Family	3	-3126.41	6258.82	14	6274.39	14
Pareto Type 2	2	-3116.53	6237.06	7	6247.44	6
Truncated Exponential Gaussian	3	-3699.95	7405.89	29	7421.47	29
Truncated Johnson's SU	4	-3111.86	6231.73	5	6252.49	7
Truncated Logistic	2	-3699.95	7403.89	28	7414.28	28
Truncated Normal	2	-3992.01	7988.01	30	7998.39	30
Truncated Power Exponential	3	-3119.03	6244.07	9	6259.64	9
Truncated Skew t Type 5	4	-3103.56	6215.12	2	6235.89	3
Truncated t Family	3	-3106.72	6219.44	3	6235.02	2
Weibull	2	-3265.47	6534.94	22	6545.32	22

Supplementary table A.2: `AutoBi`, female claimants: log-likelihood, AIC, and BIC for 30 competing models, along with rankings based on these criteria.

Model (**females**)	# par.	Log-likelihood	AIC	Rank (AIC)	BIC	Rank (BIC)
Box-Cox Cole and Green	3	-1740.27	3486.54	16	3500.37	16
Box-Cox Power Exponential	4	-1791.58	3591.16	20	3609.60	20
Box-Cox t	4	-1716.27	3440.54	1	3458.98	1
Burr	3	-1732.08	3470.16	11	3483.99	12
Dagum (Burr III)	3	-1726.68	3459.36	9	3473.19	8
Exponential	1	-2097.46	4196.92	27	4201.53	27
Gamma	2	-1928.98	3861.96	24	3871.18	24
Generalized Beta type 2	4	-1744.77	3497.54	19	3515.98	19
Generalized Gamma	3	-1740.64	3487.28	17	3501.11	17
Generalized Inverse Gaussian	3	-1852.56	3711.11	23	3724.94	23
Generalized Pareto	2	-1724.63	3453.25	6	3462.47	4
Inverse Gamma	2	-1968.63	3941.25	26	3950.47	26
Inverse Gaussian	2	-1944.84	3893.68	25	3902.90	25
Log-Gumbel	2	-1812.33	3628.66	21	3637.88	21
Log-Johnson's SU	4	-1717.57	3443.15	2	3461.59	3
Log-Logistic	2	-1733.29	3470.58	12	3479.80	11
Log-Normal	2	-1744.58	3493.16	18	3502.38	18
Log-Power Exponential	3	-1733.12	3472.24	13	3486.07	13
Log-Skew Normal Type 2	3	-1735.11	3476.21	15	3490.04	15
Log-Skew t Type 5	4	-1722.53	3453.07	5	3471.50	7
Log-t Family	3	-1733.75	3473.51	14	3487.34	14
Pareto Type 2	2	-1724.63	3453.25	7	3462.47	5
Truncated Exponential Gaussian	3	-2097.46	4200.92	29	4214.75	29
Truncated Johnson's SU	4	-1725.18	3458.36	8	3476.80	10
Truncated Logistic	2	-2097.46	4198.92	28	4208.14	28
Truncated Normal	2	-2277.52	4559.03	30	4568.25	30
Truncated Power Exponential	3	-1726.87	3459.74	10	3473.57	9
Truncated Skew t Type 5	4	-1718.17	3444.34	3	3462.78	6
Truncated t Family	3	-1719.73	3445.46	4	3459.28	2
Weibull	2	-1812.33	3628.66	22	3637.88	22

Supplementary table A.3: `AutoBi`, male claimants: log-likelihood, AIC, and BIC for 30 competing models, along with rankings based on these criteria.

Model (**males**)	# par.	Log-likelihood	AIC	Rank (AIC)	BIC	Rank (BIC)
Box-Cox Cole and Green	3	-1394.53	2795.05	17	2808.17	16
Box-Cox Power Exponential	4	-1433.81	2875.62	21	2893.12	21
Box-Cox t	4	-1391.02	2790.03	11	2807.52	15
Burr	3	-1391.70	2789.40	10	2802.52	10
Dagum (Burr III)	3	-1391.00	2788.00	8	2801.12	7
Exponential	1	-1601.03	3204.05	27	3208.42	27
Gamma	2	-1509.41	3022.81	26	3031.56	26
Generalized Beta type 2	4	-1394.63	2797.26	19	2814.75	19
Generalized Gamma	3	-1394.53	2795.06	18	2808.18	17
Generalized Inverse Gaussian	3	-1428.57	2863.14	20	2876.26	20
Generalized Pareto	2	-1391.53	2787.07	5	2795.81	3
Inverse Gamma	2	-1482.38	2968.75	25	2977.50	25
Inverse Gaussian	2	-1453.05	2910.09	24	2918.84	24
Log-Gumbel	2	-1452.65	2909.29	22	2918.04	22
Log-Johnson's SU	4	-1388.30	2784.60	4	2802.10	9
Log-Logistic	2	-1391.80	2787.60	7	2796.34	5
Log-Normal	2	-1394.60	2793.19	16	2801.94	8
Log-Power Exponential	3	-1389.09	2784.18	3	2797.30	6
Log-Skew Normal Type 2	3	-1392.66	2791.32	14	2804.44	13
Log-Skew t Type 5	4	-1390.52	2789.03	9	2806.52	14
Log-t Family	3	-1392.18	2790.36	12	2803.48	11
Pareto Type 2	2	-1391.53	2787.07	6	2795.81	4
Truncated Exponential Gaussian	3	-1601.03	3208.05	29	3221.17	29
Truncated Johnson's SU	4	-1392.08	2792.15	15	2809.64	18
Truncated Logistic	2	-1601.02	3206.05	28	3214.80	28
Truncated Normal	2	-1644.24	3292.48	30	3301.23	30
Truncated Power Exponential	3	-1392.54	2791.07	13	2804.19	12
Truncated Skew t Type 5	4	-1384.89	2777.77	1	2795.27	2
Truncated t Family	3	-1386.54	2779.08	2	2792.20	1
Weibull	2	-1452.65	2909.29	23	2918.04	23

Supplementary table A.4: `AutoBi`, total claims: VaR, difference (in percentage) with respect to the empirical VaR and ranking induced by the difference for the competing models using total claims. 95% and 99% levels are considered.

Model (**total claims**)	VaR 95%	Diff. %	Rank	VaR 99%	Diff.	Rank
Empirical	14.87			68.05		
Box-Cox Cole and Green	18.44	24.02	15	45.81	-32.68	12
Box-Cox Power Exponential	13.84	-6.90	7	40.40	-40.63	20
Box-Cox t	15.11	1.65	2	47.87	-29.65	6
Burr	4.49	-69.81	26	13.73	-79.82	28
Dagum (Burr III)	286.15	1824.76	30	2178.04	3100.82	29
Exponential	17.87	20.22	14	27.48	-59.62	25
Gamma	22.68	52.56	24	38.96	-42.75	21
Generalized Beta type 2	19.80	33.15	20	54.27	-20.24	3
Generalized Gamma	18.53	24.61	16	46.25	-32.02	11
Generalized Inverse Gaussian	26.35	77.25	28	53.68	-21.11	5
Generalized Pareto	16.52	11.14	9	44.10	-35.19	15
Inverse Gamma	125.42	743.61	29	3153.72	4534.68	30
Inverse Gaussian	26.26	76.64	27	91.08	33.85	13
Log-Gumbel	19.50	31.18	17	37.83	-44.41	22
Log-Johnson's SU	14.36	-3.38	4	46.90	-31.07	9
Log-Logistic	20.52	38.02	22	78.66	15.59	2
Log-Normal	19.70	32.50	19	53.75	-21.02	4
Log-Power Exponential	23.76	59.83	25	92.88	36.50	16
Log-Skew Normal Type 2	17.00	14.37	11	40.76	-40.10	19
Log-Skew t Type 5	15.52	4.42	6	47.18	-30.67	8
Log-t Family	19.89	33.76	21	74.45	9.41	1
Pareto Type 2	16.52	11.14	10	44.10	-35.19	14
Truncated Exponential Gaussian	17.87	20.22	13	27.48	-59.62	26
Truncated Johnson's SU	15.36	3.31	3	42.84	-37.04	17
Truncated Logistic	17.87	20.22	12	27.47	-59.62	27
Truncated Normal	21.43	44.12	23	32.46	-52.30	24
Truncated Power Exponential	16.38	10.20	8	41.96	-38.33	18
Truncated Skew t Type 5	15.05	1.21	1	47.63	-30.00	7
Truncated t Family	15.47	4.07	5	46.47	-31.71	10
Weibull	19.50	31.18	18	37.83	-44.41	23

Supplementary table A.5: `AutoBi`, female claimants: VaR, difference (in percentage) with respect to the empirical VaR and ranking induced by the difference for the competing models using the female claimants data. 95% and 99% levels are considered.

Model (**females)**	VaR 95%	Diff. %	Rank	VaR 99%	Diff.	Rank
Empirical	13.70			57.95		
Box-Cox Cole and Green	17.84	30.21	12	42.97	-25.84	6
Box-Cox Power Exponential	13.44	-1.88	2	38.33	-33.85	16
Box-Cox t	14.36	4.83	3	43.04	-25.73	5
Burr	4.24	-69.05	23	12.68	-78.12	28
Dagum (Burr III)	212.06	1447.79	30	1404.28	2323.34	29
Exponential	18.61	35.86	15	28.62	-50.62	24
Gamma	23.99	75.12	25	41.56	-28.29	11
Generalized Beta type 2	19.78	44.36	20	54.78	-5.46	2
Generalized Gamma	18.00	31.36	13	43.71	-24.57	4
Generalized Inverse Gaussian	27.65	101.80	28	55.89	-3.56	1
Generalized Pareto	15.77	15.11	8	40.64	-29.88	13
Inverse Gamma	172.23	1157.09	29	5766.62	9851.37	30
Inverse Gaussian	26.79	95.57	27	101.87	75.80	27
Log-Gumbel	19.49	42.27	18	38.16	-34.14	17
Log-Johnson's SU	13.51	-1.40	1	41.69	-28.06	10
Log-Logistic	20.56	50.09	22	79.29	36.82	20
Log-Normal	19.73	44.01	19	54.39	-6.14	3
Log-Power Exponential	23.97	74.96	24	95.54	64.87	26
Log-Skew Normal Type 2	16.29	18.86	11	38.04	-34.36	19
Log-Skew t Type 5	14.51	5.89	5	42.08	-27.39	8
Log-t Family	20.18	47.32	21	81.70	40.99	22
Pareto Type 2	15.77	15.11	9	40.64	-29.88	12
Truncated Exponential Gaussian	18.61	35.86	14	28.62	-50.62	25
Truncated Johnson's SU	15.67	14.41	7	40.47	-30.16	14
Truncated Logistic	18.62	35.87	16	28.62	-50.62	23
Truncated Normal	24.31	77.41	26	35.93	-38.00	21
Truncated Power Exponential	15.83	15.53	10	38.68	-33.24	15
Truncated Skew t Type 5	14.38	4.93	4	42.67	-26.36	7
Truncated t Family	14.76	7.75	6	41.98	-27.55	9
Weibull	19.49	42.27	17	38.16	-34.14	18

Supplementary table A.6: `AutoBi`, male claimants: VaR, difference (in percentage) with respect to the empirical VaR and ranking induced by the difference for the competing models using the male claimants data. 95% and 99% levels are considered.

Model (**males**)	VaR 95%	Diff. %	Rank	VaR 99%	Diff.	Rank
Empirical	18.59			75.37		
Box-Cox Cole and Green	19.35	4.11	3	50.94	-32.42	14
Box-Cox Power Exponential	14.59	-21.51	24	43.88	-41.78	20
Box-Cox t	17.72	-4.70	7	54.74	-27.38	5
Burr	4.87	-73.80	28	15.47	-79.47	28
Dagum (Burr III)	439.30	2263.29	30	4082.49	5316.26	30
Exponential	16.93	-8.90	16	26.03	-65.46	26
Gamma	21.03	13.11	20	35.71	-52.62	23
Generalized Beta type 2	19.72	6.07	13	53.43	-29.12	8
Generalized Gamma	19.36	4.17	4	51.01	-32.32	13
Generalized Inverse Gaussian	24.86	33.71	26	52.59	-30.23	12
Generalized Pareto	17.50	-5.87	11	48.75	-35.32	17
Inverse Gamma	74.61	301.40	29	1179.95	1465.45	29
Inverse Gaussian	25.06	34.83	27	76.34	1.29	1
Log-Gumbel	19.45	4.63	6	37.22	-50.62	21
Log-Johnson's SU	15.70	-15.53	23	53.26	-29.34	9
Log-Logistic	20.46	10.08	18	77.86	3.30	2
Log-Normal	19.63	5.63	10	52.85	-29.88	10
Log-Power Exponential	23.24	25.04	25	87.45	16.03	4
Log-Skew Normal Type 2	17.92	-3.59	1	44.40	-41.09	19
Log-Skew t Type 5	17.14	-7.79	14	53.55	-28.96	7
Log-t Family	19.56	5.21	9	65.35	-13.30	3
Pareto Type 2	17.50	-5.87	12	48.75	-35.32	18
Truncated Exponential Gaussian	16.93	-8.90	17	26.03	-65.46	27
Truncated Johnson's SU	17.69	-4.81	8	49.09	-34.87	15
Truncated Logistic	16.93	-8.90	15	26.03	-65.46	25
Truncated Normal	21.24	14.29	21	32.23	-57.24	24
Truncated Power Exponential	17.85	-3.99	2	49.07	-34.89	16
Truncated Skew t Type 5	15.91	-14.43	22	54.63	-27.52	6
Truncated t Family	16.39	-11.81	19	52.67	-30.12	11
Weibull	19.45	4.63	5	37.22	-50.62	22

Appendix B. Supplementary tables associated to Section 5.2

Supplementary table B.1: `ausprivauto0405`, total claims: log-likelihood, AIC, and BIC for 22 competing models, along with rankings based on these criteria. ZA stands for "zero-adjusted". * indicates a distribution for which the algorithm did not converge.

Model (**total claims**)	# par.	Log-likelihood	AIC	Rank (AIC)	BIC	Rank (BIC)
ZA Box-Cox t	5	-34158.81	68327.60	7	68373.20	7
ZA Exponential	2	-35392.85	70789.70	19	70807.90	19
ZA Gamma	3	-35252.01	70510.00	18	70537.40	18
ZA Generalized Gamma	4	-33776.72	67561.40	1	67597.90	1
ZA Generalized Inverse Gaussian	4	-34140.59	68289.20	6	68325.70	5
ZA Generalized Pareto	3	-34758.95	69523.90	15	69551.30	15
ZA Inverse Gaussian	3	-34180.92	68367.80	8	68395.20	8
ZA Log-Gumbel	3	-35080.69	70167.40	16	70194.80	16
ZA Log-Johnson's SU	5	-34126.27	68262.50	4	68308.20	4
ZA Log-Logistic	3	-34552.76	69111.50	13	69138.90	13
ZA Log-Normal	3	-34441.25	68888.50	11	68915.90	11
ZA Log-Power Exponential	4	-34349.05	68706.10	10	68742.60	10
ZA Log-Skew Normal Type 2*	4	-33987.49	67983.00	2	68019.50	2
ZA Log-Skew t Type 5	5	-34186.05	68382.10	9	68427.70	9
ZA Log-t Family	4	-34441.25	68890.50	12	68927.00	12
ZA Truncated Exponential Gaussian	4	-35393.24	70794.50	21	70831.00	21
ZA Truncated Logistic	3	-35393.95	70793.90	20	70821.30	20
ZA Truncated Normal*	3	-35597.39	71200.80	22	71228.20	22
ZA Truncated Power Exponential*	4	-34064.27	68136.50	3	68173.00	3
ZA Truncated Skew t Type 5	5	-34138.65	68287.30	5	68332.92	6
ZA Truncated t Family	4	-34706.43	69420.90	14	69457.40	14
ZA Weibull	3	-35080.69	70167.40	17	70194.80	17

Supplementary table B.2: `ausprivauto0405`, female claimants: log-likelihood, AIC, and BIC for 22 competing models, along with rankings based on these criteria. ZA stands for "zero-adjusted". * indicates a distribution for which the algorithm did not converge.

Model (**females**)	# par.	Log-likelihood	AIC	Rank (AIC)	BIC	Rank (BIC)
ZA Box-Cox t	5	-19408.57	38827.10	6	38869.90	7
ZA Exponential	2	-20030.04	40064.10	19	40081.20	19
ZA Gamma	3	-19977.27	39960.50	18	39986.20	18
ZA Generalized Gamma	4	-19175.88	38359.80	1	38394.00	1
ZA Generalized Inverse Gaussian	4	-19392.71	38793.40	4	38827.70	3
ZA Generalized Pareto	3	-19746.14	39498.30	15	39524.00	15
ZA Inverse Gaussian	3	-19411.05	38828.10	7	38853.80	6
ZA Log-Gumbel	3	-19900.89	39807.80	16	39833.50	16
ZA Log-Johnson's SU	5	-19388.50	38787.00	3	38829.80	4
ZA Log-Logistic	3	-19627.93	39261.90	13	39287.60	13
ZA Log-Normal	3	-19556.73	39119.50	11	39145.10	11
ZA Log-Power Exponential	4	-19484.08	38976.20	10	39010.40	10
ZA Log-Skew Normal Type 2*	4	-19290.14	38588.30	2	38622.50	2
ZA Log-Skew t Type 5	5	-19424.50	38859.00	9	38901.80	9
ZA Log-t Family	4	-19556.73	39121.50	12	39155.70	12
ZA Truncated Exponential Gaussian	4	-20377.49	40763.00	22	40797.20	22
ZA Truncated Logistic	3	-20030.79	40067.60	20	40093.30	20
ZA Truncated Normal*	3	-20132.50	40271.00	21	40296.70	21
ZA Truncated Power Exponential*	4	-19414.26	38836.50	8	38870.80	8
ZA Truncated Skew t Type 5	5	-19394.58	38799.20	5	38842.00	5
ZA Truncated t Family	4	-19724.19	39456.40	14	39490.60	14
ZA Weibull	3	-19900.89	39807.80	17	39833.50	17

Supplementary table B.3: `ausprivauto0405`, male claimants: log-likelihood, AIC, and BIC for 22 competing models, along with rankings based on these criteria. ZA stands for "zero-adjusted". * indicates a distribution for which the algorithm did not converge.

Model (**males**)	# par.	Log-likelihood	AIC	Rank (AIC)	BIC	Rank (BIC)
ZA Box-Cox t	5	-14747.99	29506.00	6	29547.40	6
ZA Exponential	2	-15343.23	30690.50	18	30707.00	18
ZA Gamma	3	-15255.56	30517.10	17	30542.00	17
ZA Generalized Gamma	4	-14600.08	29208.20	1	29241.30	1
ZA Generalized Inverse Gaussian	4	-14742.73	29493.50	5	29526.60	4
ZA Generalized Pareto	3	-15008.07	30022.10	14	30047.00	14
ZA Inverse Gaussian	3	-14762.77	29531.50	8	29556.40	7
ZA Log-Gumbel	3	-15165.99	30338.00	16	30362.80	16
ZA Log-Johnson's SU	5	-14734.78	29479.60	3	29521.00	3
ZA Log-Logistic	3	-14921.85	29849.70	12	29874.60	12
ZA Log-Normal	3	-14879.39	29764.80	10	29789.60	10
ZA Log-Power Exponential	4	-14849.23	29706.50	9	29739.60	9
ZA Log-Skew Normal Type 2*	4	-14690.12	29388.20	2	29421.40	2
ZA Log-Skew t Type 5	5	-14760.67	29531.30	7	29572.80	8
ZA Log-t Family	4	-14879.39	29766.80	11	29799.90	11
ZA Truncated Exponential Gaussian	4	-15345.63	30699.30	20	30732.40	20
ZA Truncated Logistic	3	-15343.88	30693.80	19	30718.60	19
ZA Truncated Normal*	3	-15430.47	30866.90	21	30891.80	21
ZA Truncated Power Exponential*	4	-15011.99	30032.00	15	30065.10	15
ZA Truncated Skew t Type 5	5	-14741.32	29492.65	4	29534.07	5
ZA Truncated t Family	4	-14979.21	29966.40	13	29999.60	13
ZA Weibull	3	-20307.65	40621.31	22	40646.20	22

Supplementary table B.4: `ausprivauto0405`, total claims: VaR, difference (in percentage) with respect to the empirical VaR and ranking induced by the difference for the competing models using total claims. 95% and 99% levels are considered.

Model (**total claims**)	VaR 95%	Diff. %	Rank	VaR 99%	Diff.	Rank
Empirical	3.54			36.25		
ZA Box-Cox t	3.76	6.21	7	31.07	-14.31	19
ZA Exponential	6.24	76.33	20	38.67	6.67	8
ZA Gamma	4.52	27.76	17	40.43	11.53	12
ZA Generalized Gamma	3.12	-11.89	11	38.11	5.13	3
ZA Generalized Inverse Gaussian	4.06	14.73	12	31.57	-12.90	17
ZA Generalized Pareto	3.60	1.75	3	34.29	-5.42	4
ZA Inverse Gaussian	4.19	18.52	14	35.67	-1.62	1
ZA Log-Gumbel	3.80	7.55	8	38.78	6.97	10
ZA Log-Johnson's SU	3.75	6.13	6	32.03	-11.65	13
ZA Log-Logistic	4.12	16.38	13	28.52	-21.32	22
ZA Log-Normal	4.32	22.06	15	31.64	-12.73	16
ZA Log-Power Exponential	4.60	29.88	18	41.35	14.06	18
ZA Log-Skew Normal Type 2	3,72	5.06	4	34.48	-4.89	2
ZA Log-Skew t Type 5	3.92	10.69	10	30.66	-15.43	20
ZA Log-t Family	4.32	22.06	16	31.64	-12.73	15
ZA Truncated Exponential Gaussian	6.24	76.51	21	38.71	6.76	9
ZA Truncated Logistic	6.23	76.20	19	38.62	6.53	7
ZA Truncated Normal	6.80	92.27	22	40.72	12.34	14
ZA Truncated Power Exponential	3.54	0.04	1	38.40	5.91	5
ZA Truncated Skew t Type 5	3.49	-1.26	2	33.99	-6.24	6
ZA Truncated t Family	3.75	6.12	5	30.62	-15.55	21
ZA Weibull	3.80	7.55	9	38.78	6.97	11

Supplementary table B.5: `ausprivauto0405`, female claimants: VaR, difference (in percentage) with respect to the empirical VaR and ranking induced by the difference for the competing models using the female claims. 95% and 99% levels are considered.

Model (**females**)	VaR 95%	Diff. %	Rank	VaR 99%	Diff.	Rank
Empirical	3.54			34.32		
ZA Box-Cox t	3.70	4.68	6	29.65	-13.60	17
ZA Exponential	5.86	65.68	20	35.70	4.02	4
ZA Gamma	4.56	28.76	17	37.06	7.99	11
ZA Generalized Gamma	3.11	-12.18	11	36.04	5.01	9
ZA Generalized Inverse Gaussian	4.03	13.78	12	30.09	-12.32	14
ZA Generalized Pareto	3.61	2.15	2	32.53	-5.20	10
ZA Inverse Gaussian	4.15	17.38	14	33.17	-3.34	2
ZA Log-Gumbel	3.89	9.87	10	36.03	4.98	8
ZA Log-Johnson's SU	3.70	4.63	5	30.62	-10.77	13
ZA Log-Logistic	4.10	15.78	13	27.29	-20.47	22
ZA Log-Normal	4.29	21.25	15	29.92	-12.81	16
ZA Log-Power Exponential	4.62	30.45	18	40.85	19.03	21
ZA Log-Skew Normal Type 2	3.66	3.56	4	32.92	-4.06	5
ZA Log-Skew t Type 5	3.88	9.63	8	29.24	-14.80	19
ZA Log-t Family	4.29	21.25	16	29.92	-12.81	15
ZA Truncated Exponential Gaussian	7.50	112.04	22	40.14	16.96	20
ZA Truncated Logistic	5.86	65.51	19	35.63	3.83	3
ZA Truncated Normal	6.33	79.02	21	37.14	8.22	12
ZA Truncated Power Exponential	3.54	-0.03	1	35.19	2.54	1
ZA Truncated Skew t Type 5	3.42	-3.37	3	32.77	-4.51	6
ZA Truncated t Family	3.75	6.12	7	29.28	-14.69	18
ZA Weibull	3.89	9.87	9	36.03	4.98	7

Supplementary table B.6: `ausprivauto0405`, male claims: VaR, difference (in percentage) with respect to the empirical VaR and ranking induced by the difference for the competing models using the male claims. 95% and 99% levels are considered.

Model (**males**)	VaR 95%	Diff. %	Rank	VaR 99%	Diff.	Rank
Empirical	3.60			38.06		
ZA Box-Cox t	3.82	6.33	8	32.99	-13.31	16
ZA Exponential	6.71	86.45	20	42.59	11.91	14
ZA Gamma	4.48	24.42	17	44.85	17.84	20
ZA Generalized Gamma	3.13	-13.05	11	40.19	5.59	5
ZA Generalized Inverse Gaussian	4.11	14.29	12	33.64	-11.62	12
ZA Generalized Pareto	3.59	-0.25	2	36.66	-3.67	3
ZA Inverse Gaussian	4.25	18.06	14	38.83	2.04	2
ZA Log-Gumbel	3.72	3.54	4	42.31	11.16	10
ZA Log-Johnson's SU	3.83	6.47	9	33.90	-10.91	9
ZA Log-Logistic	4.14	15.20	13	30.23	-20.55	22
ZA Log-Normal	4.36	21.13	15	33.98	-10.71	8
ZA Log-Power Exponential	4.62	28.47	18	43.58	14.51	18
ZA Log-Skew Normal Type 2	3.81	5.88	7	36.56	-3.93	4
ZA Log-Skew t Type 5	3.98	10.57	10	32.62	-14.28	17
ZA Log-t Family	4.36	21.13	16	33.98	-10.71	7
ZA Truncated Exponential Gaussian	6.73	86.97	21	42.70	12.19	15
ZA Truncated Logistic	6.70	86.21	19	42.50	11.68	13
ZA Truncated Normal	7.34	104.13	22	45.24	18.88	21
ZA Truncated Power Exponential	3.50	-2.64	3	38.27	0.56	1
ZA Truncated Skew t Type 5	3.60	-0.02	1	35.76	-6.03	6
ZA Truncated t Family	3.76	4.43	6	32.53	-14.52	19
ZA Weibull	3.72	3.56	5	42.31	11.18	11

Appendix C. Supplementary figures

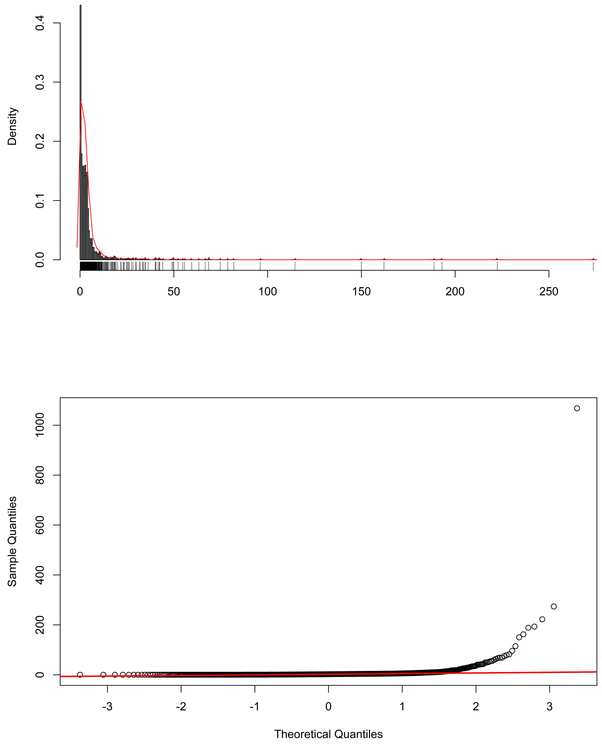

Supplementary figure C.1: Histogram (kernel density estimate in red) and normal Q-Q plot for the total losses of the `AutoBi` data. The horizontal axis of the histogram is restricted to 250.

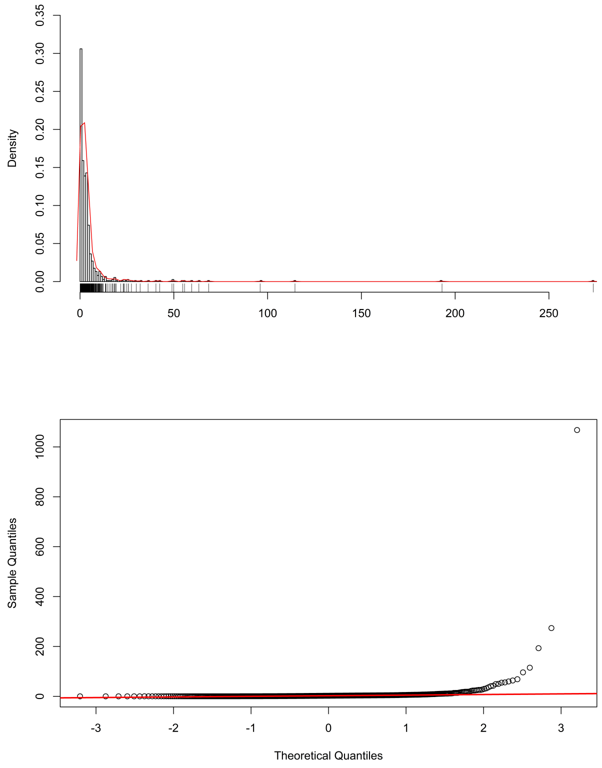

Supplementary figure C.2: Histogram (kernel density estimate in red) and normal Q-Q plot for the losses of female claimants of the `AutoBi` data. The horizontal axis of the histogram is restricted to 250.

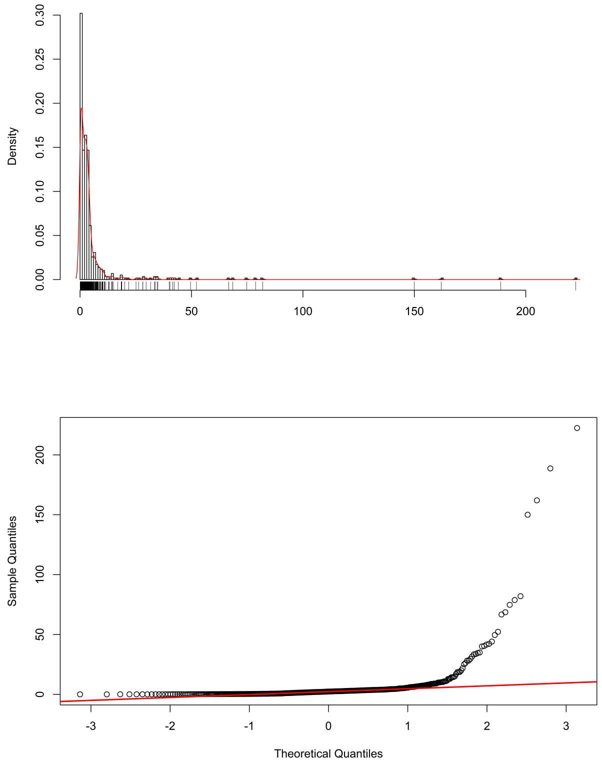

Supplementary figure C.3: Histogram (kernel density estimate in red) and normal Q-Q plot for the losses of male claimants of the `AutoBi` data. The horizontal axis of the histogram is not restricted.

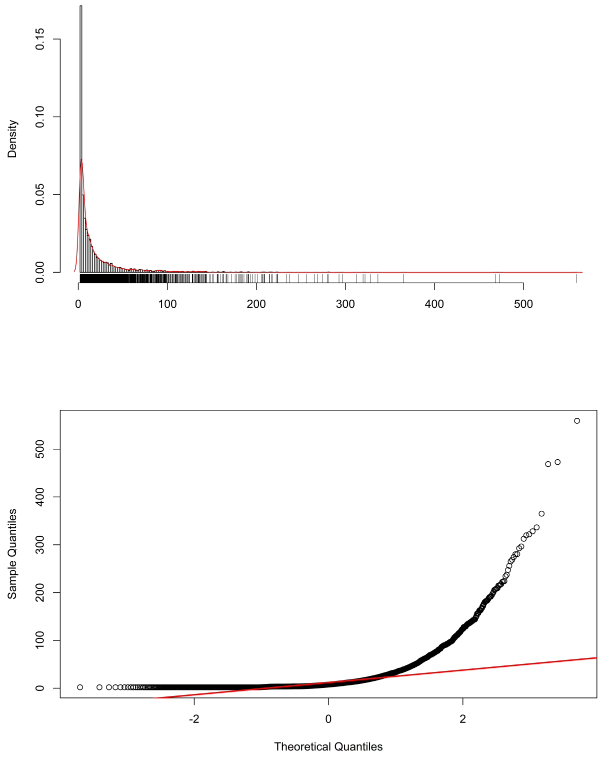

Supplementary figure C.4: Histogram (kernel density estimate in red) and normal Q-Q plot for the total losses of the `ausprivauto0405` data. Zeros are excluded.

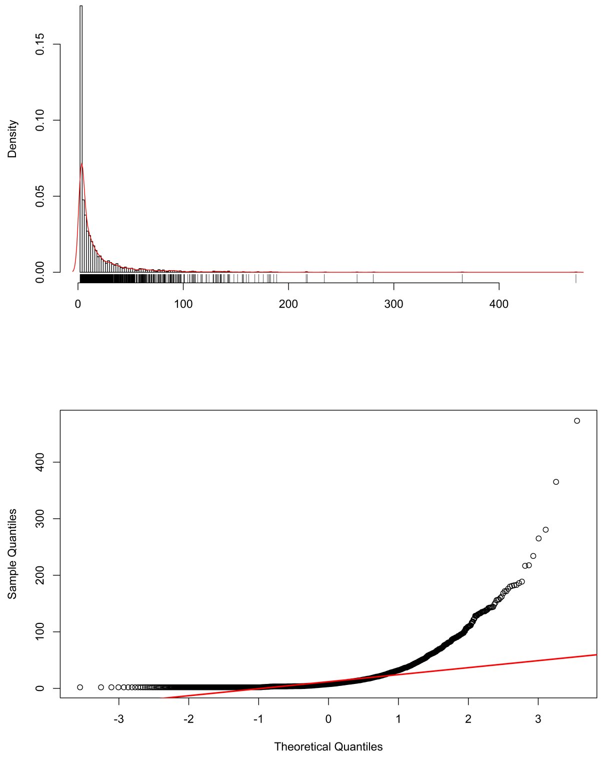

Supplementary figure C.5: Histogram (kernel density estimate in red) and normal Q-Q plot for the losses of female claimants of the `ausprivauto0405` data. Zeros are excluded.

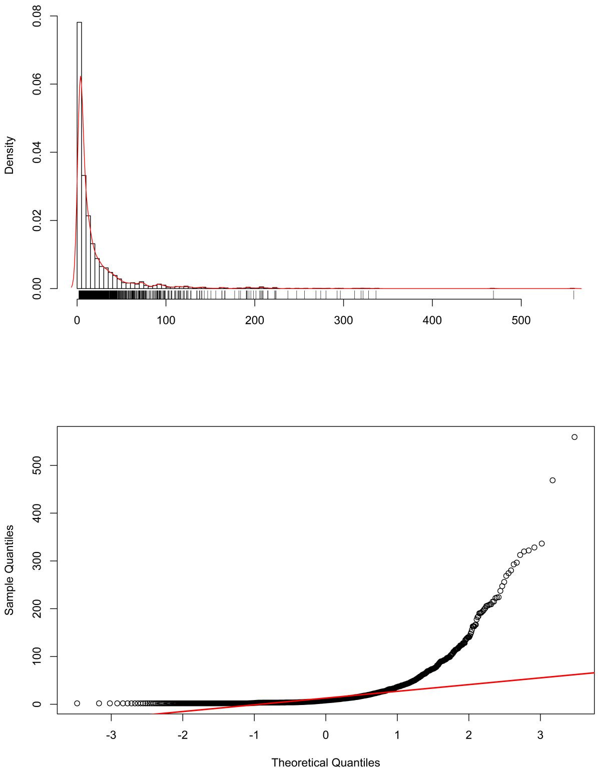

Supplementary figure C.6: Histogram (kernel density estimate in red) and normal Q-Q plot for the losses of male claimants of the `ausprivauto0405` data. Zeros are excluded.
